# Modeling the Mutational Effects on Biochemical Phenotypes of SARS-CoV-2 Using Molecular Fields

**DOI:** 10.3390/biom15111538

**Published:** 2025-10-31

**Authors:** Baifan Wang, Zhen Xi

**Affiliations:** State Key Laboratory of Elemento-Organic Chemistry, Department of Chemical Biology, National Pesticide Engineering Research Center (Tianjin), Nankai University, Tianjin 300071, China

**Keywords:** SARS-CoV-2, MB-QSAR, predictive modeling, protein–protein interaction, hACE2 binding, antibody escape, mutational effects

## Abstract

The ongoing evolution of SARS-CoV-2 has given rise to variants with enhanced transmissibility and pathogenicity, many of which harbor mutations in the receptor-binding domain (RBD) of the viral spike protein. These mutations often confer increased viral fitness and immune evasion by modulating interactions with the human ACE2 receptor (hACE2) and escaping neutralizing antibodies. Accurate prediction of the functional consequences of such mutations—particularly their effects on receptor binding and antibody escape—is critical for assessing the public health threat posed by emerging variants. In this study, we apply a Mutation-dependent Biomacromolecular Quantitative Structure–Activity Relationship (MB-QSAR) framework to quantitatively model the biochemical phenotypes of RBD variants. Trained on comprehensive deep mutational scanning (DMS) datasets, our models exhibit strong predictive performance, achieving correlation coefficients (r^2^) exceeding 0.8 for hACE2 binding affinity and 0.7 for antibody neutralization escape. Importantly, the MB-QSAR approach generalizes well to multi-mutant variants and currently circulating lineages. Structural analysis based on model-derived interaction profiles offers mechanistic insights into key RBD–ACE2 and RBD–antibody interfaces, helping the rational design of broadly protective vaccines and therapeutics. This work establishes MB-QSAR as a rapid, accurate, and interpretable tool for the prediction of protein–protein interaction and forecasting viral adaptation, thereby facilitating early risk assessment of novel SARS-CoV-2 variants.

## 1. Introduction

The COVID-19 pandemic, caused by the SARS-CoV-2 virus, remains a critical global public health crisis, with profound socioeconomic and ecological consequences [1,2]. The high mutation rate of SARS-CoV-2, driven by its RNA-based genome, has led to the emergence of numerous variants, many of which exhibit enhanced transmissibility, immune evasion, or altered pathogenicity [3,4]. Among these, Omicron subvariants such as BA.2.75, BQ.1.1, XBB, XBB.1.5, JN.1, and KP.3 have successively dominated the pandemic landscape [5,6,7]. These variants frequently harbor mutations in the spike protein’s receptor-binding domain (RBD), a region critical for viral entry via interaction with the human angiotensin-converting enzyme 2 (hACE2) receptor. Mutations in the RBD can enhance binding affinity to hACE2 and impair antibody-mediated neutralization, thereby undermining the efficacy of current vaccines and therapeutic antibodies [8,9,10,11,12].

The molecular architecture of the RBD-hACE2 interface (residues 331–524) has been extensively characterized, with key residues governing both receptor engagement and immune recognition [13,14,15,16]. This interaction is a critical determinant of viral infectivity and a primary target for host immune responses. Structural studies reveal that the RBD-hACE2 interface comprises polar and hydrophobic interactions, with mutations in this region directly influencing binding affinity and immune evasion [16]. Notably, the RBD is the target of over 90% of neutralizing antibodies elicited by infection or vaccination [17,18,19,20], making it a hotspot for antigenic drift under immune pressure.

Emerging variants often carry RBD mutations that confer increased transmissibility [21], alter infectivity [22,23], or escape neutralizing antibodies [22,23]. It was shown that the RBD of spike protein is the target for more than 90% of the neutralization antibodies generated by humoral response [24], which exerts significant selection pressure on RBD. Thus, RBD is likely the most susceptible target to antigenic escape due to amino acid mutation. Therefore, RBD-adapted mutations that can increase its binding affinity with hACE2 and/or adversely affect antibody neutralization have been extensively mapped by high-throughput mutational studies [25,26,27]. For example, the K417N/T, L452R, E484K, and N501Y mutation of RBDs found in Variants of Concern (VOCs) can increase binding affinity towards hACE2 while eliciting immune escape and reduced neutralization of RBD-targeting antibodies [28,29,30,31,32,33].

It was found that approximately 1 million sequences had at least one mutation in the RBD, in which 92% involved a mutation in the RBD that improves binding to hACE2 as measured by deep mutational scanning [27,34]. This implies that binding-improving mutations in the RBD are at least 11-fold enriched among circulating variants [34]. In addition, analysis of SARS-CoV-2 genome sequences suggests that there are thousands of mutations whose biological and health implications are unknown (CoV-GLUE database). Hence, continued surveillance and methods to accurately predict affinity gains of the RBD-hACE2 binding event and/or immune escape due to mutation in the RBD remain important.

Computational methods can help assess the mechanistic role of the mutation occurring in circulating viral variants and also predict potentially problematic mutations that have not been identified so far. Zhou et al. suggested that the N439K variant in spike protein displays a higher binding affinity to hACE2 and resistance to the antibody REGN10987, using molecular dynamics (MD) was validated by experimental evidence [35]. Chen et al. used a machine learning (ML) model to quantify the binding free energy changes in RBDs from several existing SARS-CoV-2 variants [36]. Laurini et al. performed a computational mutagenesis of the RBD-ACE2 interface residues and assessed changes in binding energies using MD simulations and validated using experimental data [37]. Chen et al. implemented a neural network to predict the binding affinity between RBD and hACE2 variants using the decomposed energy terms from MD simulation as descriptors [34]. Calcagnile et al. used molecular docking to predict the effects of hACE2 missense variants on the interaction with the spike protein of SARS-CoV-2 [38]. Wang et al. proposed an artificial intelligence-based framework called UniBind to predict the binding affinities between SARS-CoV-2 spike protein variants and human ACE2 [39].

However, conventional methods face critical limitations: MD simulations require prohibitive computational resources for systematic variant screening, and most models inadequately address antibody escape phenotypes. Although deep mutational scanning (DMS) provides experimental datasets for RBD-antibody interactions [12,25,26,27], laboratory validation of putative high-risk mutations remains time- and cost-intensive.

To bridge this gap, we present an extension of our Mutation-dependent Biomacromolecular Quantitative Structure-Activity Relationship (MB-QSAR) methodology [40,41,42,43,44]. Originally developed for small molecule-protein interactions, we now adapt MB-QSAR to model viral evolution by treating protein variants as “molecular ligands.” The core premise of MB-QSAR is that a missense mutation alters the physicochemical field—encompassing steric bulk, electrostatic potential, and hydrophobicity—at a protein’s interaction interface. Rather than a symbolic change, a mutation is represented as a tangible perturbation to this 3D field landscape, which directly governs molecular recognition and binding energetics. This provides an intuitive and biophysically grounded representation of a mutation’s effect. We apply this framework to two pivotal aspects of viral evolution: The quantitative prediction of RBD-hACE2 binding affinity changes and The assessment of the antibody escape potential of RBD variants. The phenotypes we predict (binding affinity and immune evasion) are ultimately determined by the compatibility of interaction fields between the viral RBD and its host partners (hACE2 or antibodies). The Comparative Molecular Field Analysis (CoMFA/CoMSIA) descriptors in MB-QSAR were originally designed to model these exact forces for small molecule binding. We extend this paradigm by treating the “ligand” as a protein variant with a localized perturbation to its surface fields. By integrating these structural descriptors with mutational landscapes, our approach enables the rapid identification of variants with enhanced infectivity or immune evasion capacity. This framework not only complements existing strategies but also provides actionable insights for preemptive vaccine design and therapeutic antibody development.

## 2. Materials and Methods

### 2.1. Dataset Preparation

We select 29 and 35 RBD residues that make direct contact with hACE2 and antibody combination at the binding interface, respectively. Depending on the availability of the data source, this gives 522 and 546 possible variants upon changing each one of the above-mentioned residues into the remaining 19 amino acids.

Experimental binding affinities of RBD variant bound to hACE2 were obtained from the deep mutagenesis study [25,27,45]. For the binding of RBD variants to ACE2, Starr et al. reported the relative apparent dissociation constant *K*_D_ ratios of variants to the wild-type (∆log_10_*K*_D, app_, referred to as relative p*K*_D_). A positive relative p*K*_D_ value for a variant implies stronger binding compared to wild-type, whereas a negative value implies weaker binding. The dataset was then divided into training and test sets (223:299) (Table S1), in which the training set was used to construct the MB-QSAR model while the test set was used to evaluate the predictive performance of the MB-QSAR model. The division of training and test sets ensured the proper distribution of the relative p*K*_D_ values and the properties of the mutated residue of the variants (Table S1, Figure S1).

We also incorporated a series of p*K*_D_ form RBD multiple mutants (e.g., in vitro evolved variants and circulating lineages like Omicron see Tables S4 and S5), performed as a separate validation step to test the model’s generalizability and its ability to extrapolate beyond its training data.

For the antibody combination, the experimental data used by the escape calculator were drawn from previously published deep mutational scanning studies against a panel of 33 neutralizing antibodies [25,46,47,48,49,50]. The deep mutational scanning measures an escape fraction for each RBD mutation against each antibody, which represents an estimate of how completely that mutation escapes antibody binding [26]. This panel was curated to represent a diverse range of epitope classes on the RBD (Class 1–4), ensuring that the aggregated escape fraction reflects a variant’s potential for broad immune evasion. The 33 antibodies used to generate the “antibody combination” escape score were selected to represent a broad spectrum of known epitope classes on the RBD. These include:

Class 1 antibodies: Target the receptor-binding motif (RBM) and often compete with ACE2 binding (e.g., LY-CoV555).

Class 2 antibodies: Also target the RBM but bind to a different footprint and are less affected by ACE2-competing mutations (e.g., LY-CoV016).

Class 3 antibodies: Bind to RBD epitopes outside the RBM and typically do not compete with ACE2 (e.g., S309).

Class 4 antibodies: Bind to a more conserved, cryptic epitope that is only exposed in the “up” RBD conformation.

By aggregating the escape fractions across this diverse panel, the resulting “escape score” serves as a robust, generalized metric for a variant’s overall potential to evade a polyclonal antibody response, rather than its escape from any single antibody. The antibodies used and their respective epitope classifications were lists in supplementary table (Table S2).

The training and test sets were partitioned according to the previous work [40,41]. First, the range of apparent p*K*_D_ values in the test set was designed to closely match that of the training set. Second, we performed K-means clustering analysis of amino acids and classified the twenty natural amino acids into four distinct groups:

Group 1: Glycine, Alanine, Valine, Leucine, Isoleucine, Serine, Threonine;

Group 2: Phenylalanine, Tryptophan, Methionine, Tyrosine;

Group 3: Cysteine, Aspartic acid, Glutamic acid, Proline;

Group 4: Histidine, Lysine, Asparagine, Glutamine, Arginine.

We ensured that each of these four clusters was represented in both the training and test sets.

Additionally, we maintained similar distributions of bioactivity data between the two sets, thereby guaranteeing that mutants in both datasets exhibit comparable structural diversity and activity profiles.

### 2.2. Modeling of the Structure of SARS-COV-2 RBD Variants

Modeller10.1 [51] was used for homology modeling to generate the structure of SARS-COV-2 RBD variants. The 3D coordinates of the SARS-CoV-2 RBD in complex with hACE2 or antibody LY-CoV016 were obtained from the Protein Data Bank entries 6M0J [16] and 7C01 [18] and used as a template, respectively. The glycan and water molecules were removed from the structure. The structure of RBD variants complexed with hACE2 was generated, and local optimization of the mutated residue region was performed. Then the coordinates of hACE2 were removed from the structure. Hydrogen atoms were added to RBD variants by SYBYL6.9. The amber charges were assigned to proteins.

### 2.3. MB-QSAR Modeling

Before the molecular field analysis, all homology-modeled RBD variants were superimposed on the wild-type. Then the CoMFA and CoMSIA molecular field parameters of each variant were calculated using SYBYL6.9. The structure of each variant was embedded in a 2.0 Å spacing lattice on the selected regions. The CoMFA fields were calculated with a distance-dependent dielectric constant (1/r), and a sp^3^ carbon atom with +1.0 charges serving as the probe atom was used to calculate the steric and electrostatic field values. An energy cutoff value of 30 kcal/mol was used for both the steric and electrostatic fields. In CoMSIA studies, five indices (steric (S), electrostatic (E), hydrophobic (H), hydrogen-bond donor (D), and hydrogen-bond acceptor (A) descriptors) were calculated with the same lattice as in the CoMFA fields calculation, using the probe atom with a radius of 1.0 Å, a charge of +1.0 and a unit hydrophobicity value. A Gaussian-type distance dependence function was used between the grid points and the atoms of the proteins.

For each studied system, the dataset was divided into a training set and a test set. MB-QSAR models were constructed based on the training set. The test set was used to evaluate the external predictability of these models. The CoMFA and CoMSIA field values were used as independent variables, while the biological data were used as dependent variables in the partial least squares (PLS) regression analyses to derive the MB-QSAR models. The cross-validation with the leave-one-out (LOO) option was carried out, and the SAMPLS method was used in CoMSIA to obtain the optimal number of components (ONC), and the ONC was used to generate the PLS regression models by non-cross-validated analysis. In the case of CoMSIA analysis, 31 analyses were carried out using the five fields separately and in all possible combinations. All the QSAR calculations were performed in SYBYL6.9. The Standard Error of Prediction (SEP) and confidence interval for the test set predictions were calculated using an in-house script.

## 3. Results

**Figure 1:**
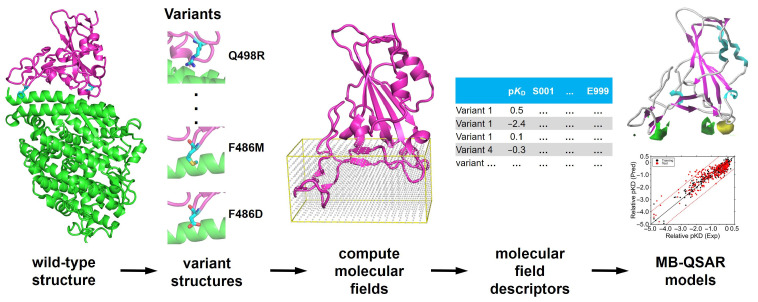
Schematic representation of the workflow for building the MB-QSAR model. The structures of a series of variants were constructed, and then the molecular field values in the region were computed using probe atoms. The PLS regression method was used to correlate the biological values and the calculated molecular field descriptors to construct the MB-QSAR models. The constructed MB-QSAR models can be used to predict the biological data of uncharacterized variants and provide molecular field view insight into the correlation between the structure and the biological data.

The MB-QSAR method is based on the small molecule 3D-QSAR methodology [52,53] but performed on a series of protein variants. In this approach, the structural information of studied protein variants was represented as 3D molecular field parameters. The region of interest of the protein variants was embedded in a grid lattice, and the molecular field parameters (such as steric and electrostatic potentials) were calculated with probe atoms. Partial least squares (PLS) regression was employed to associate the molecular field parameters and the experimentally determined properties to construct the prediction model (Figure 1).

### 3.1. Construction of MB-QSAR Models

**Figure 2:**
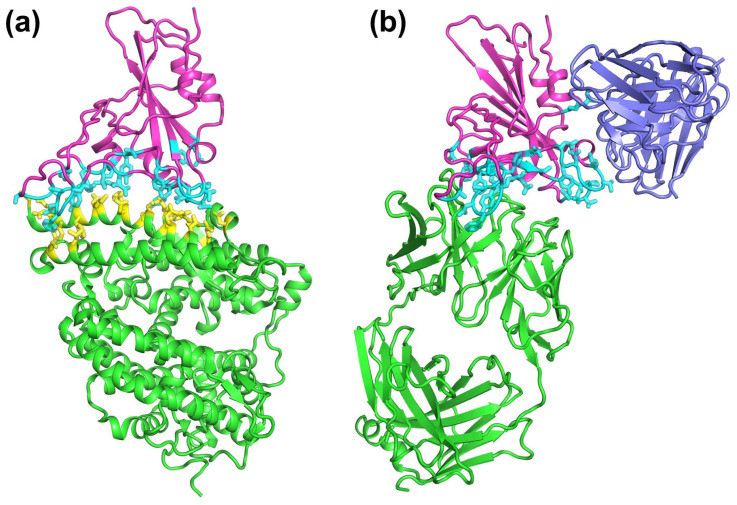
Structures of RBD bound to hACE2 and antibodies. (**a**) The crystal structure of the complex formed between RBD and hACE2 (PDBID: 6M0J). The RBD is shown as a cartoon representation in magenta, and the ACE2 is shown in green. (**b**) The crystal structure of the complex of RBD-bound antibody LY-CoV016, as well as C135 (PDBID: 7C01 and 7K8Z, respectively). The RBD is shown as a cartoon representation in magenta; the LY-CoV016 is shown in blue, respectively. Residues of the RBD variants that are involved in this study are depicted as cyan sticks. Residues of the hACE2 that are involved in the direct interaction with RBD are depicted as yellow sticks.

We employed the MB-QSAR method to investigate two biochemical properties, binding affinity between RBD variants and hACE2, and antibody escape fraction of RBD variants to the combination of 33 antibodies (Table S2). We focused on the RBD residues that make direct contact with hACE2 and the antibody combination at the binding interface, respectively (Figure 2).

The MB-QSAR models for RBD variants against hACE2 and antibody combinations with 33 antibodies were constructed (named RBD-hACE2 and RBD-antibodies, respectively). The statistical results of the MB-QSAR/CoMFA models for the studied systems are shown in Table 1. Six statistical parameters, including the *q*^2^, *ONC*, *r*^2^, *SEE*, *F*-value, and *r*^2^_pred_ value, were obtained to assess the quality of MB-QSAR models. In general, our MB-QSAR/CoMFA models for the three study systems were quite good, considering their cross-validated squared correlation coefficient *q*^2^ values were about 0.7 using 4 components and the high *r*^2^ values. The higher *F*-values and the lower *SEE* also indicated our models had higher explanatory power.

In the MB-QSAR/CoMFA models of RBD-hACE2, the contributions of the steric and electrostatic fields are approximately 55% and 45%, respectively, which implies that the steric field plays a more important role in the binding of RBD variants to hACE2. For the antibody combination system, the electrostatic field displayed a higher contribution to the MB-QSAR/CoMFA model than the steric field.

Compared to CoMFA methods, CoMSIA can utilize up to five different molecular fields (steric, electrostatic, hydrophobic, hydrogen bond donor, and hydrogen bond acceptor fields) as well as their combinations to construct QSAR models. We tested all 31 possible field combinations to generate the MB-QSAR/CoMSIA models. The field (combinations) displaying the highest *q*^2^ value and best external predictive ability (*r*^2^_pred_) were chosen as the MB-QSAR/CoMSIA models for the three study systems (Table 1).

**Figure 3:**
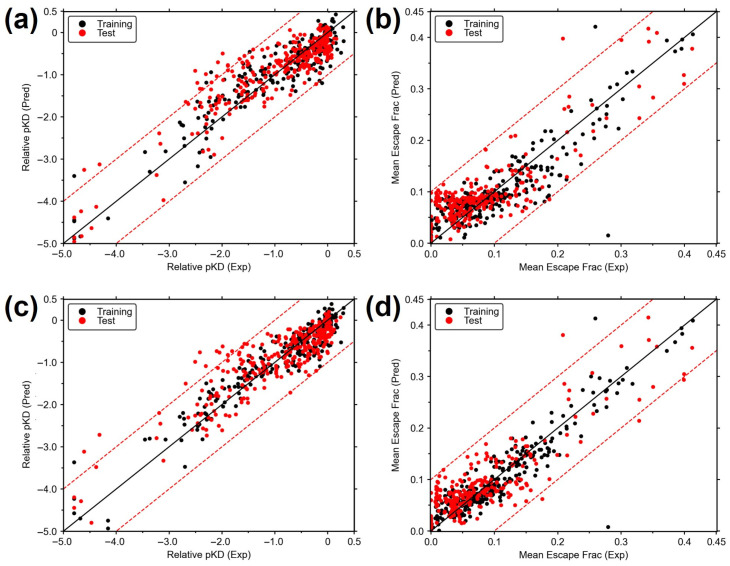
Plots of the experimental and predicted mutational effect values in the MB-QSAR CoMFA and CoMSIA models. (**a**,**b**): CoMFA model for RBD-hACE2 RBD-antibodies, respectively; (**c**,**d**): CoMSIA model for RBD-hACE2 and RBD-antibodies, respectively. The values from the training and test sets are shown in black and red dots, respectively. The black line represents the identity between the experimental and the predicted values, while the red dashed lines display 1.0 and 0.1 value errors from identity for RBD-hACE2 and RBD-antibodies, respectively.

The obtained CoMFA and CoMSIA models were used to predict the biochemical properties of the test set (Figure 3, Tables S1 and S3). For the three studied systems, the MB-QSAR models, the *r*^2^_pred_ values for the prediction of test experimental values are around or higher than 0.7 (Table 1). These results indicated high prediction accuracy for all of the MB-QSAR models.

### 3.2. Prediction of pKD of the Circulating SARS-CoV-2 Variants Using MB-QSAR Models

The currently circulating SARS-CoV-2 variants featured amino acid mutations on the RBD of spike, which could enhance the binding of RBD to hACE2 or escape the neutralization of antibodies (20). Of the underlying mutations, some were part of our training set (e.g., L452R, Y453F, S477N), whereas others (e.g., K417T, K417N, N501Y, E484K, F490S, and S494P) were not. We then test if our MB-QSAR model, which was built from a dataset composed of a single mutation of RBD, could be employed to predict the binding affinity of the RBD variants incorporating multiple mutations to hACE2.

**Figure 4:**
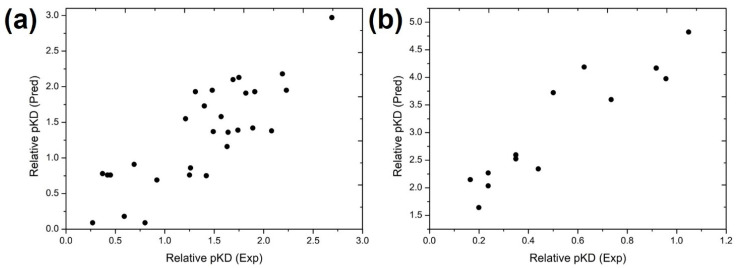
Plots of the experimental and MB-QSAR CoMSIA model predicted relative p*K*_D_ values for the RBD variants. (**a**) RBD variants from an in vitro evolution, (**b**) Omicron variants.

We first applied the MB-QSAR model to the prediction of the relative p*K*_D_ of RBD variants from an in vitro evolution that contains multiple mutations on RBD [54], as well as RBD from circulating SARS-CoV-2 variants. We found a strong correlation between the predicted and experimental relative p*K*_D_ values of RBD variants from in vitro evolution with Pearson correlation coefficients of 0.70 and 0.82 for the CoMFA model and CoMSIA models, respectively (Figure 4a and Table S4), indicating our MB-QSAR models were capable of reproducing the binding affinity of multiple mutation RBD variants towards hACE2.

Given that the MB-QSAR/CoMSIA model demonstrated superior predictive power for multi-mutant RBD variants compared to the CoMFA model, we subsequently applied the CoMSIA model to predict the p*K*_D_ values of circulating SARS-CoV-2 Omicron variants. It was shown that the RBD from most variants displayed enhanced binding to hACE2 compared to the wild-type, while our prediction correctly captured this characteristic (Figure 4b and Table S5).

### 3.3. Prediction of Antibody Escape Fraction of SARS-CoV-2 Variants

We set out to investigate whether the MB-QSAR model can be used to predict the escape fraction of RBD variants towards the neutralization of polyclonal antibodies elicited by SARS-CoV-2 vaccination or infection. By averaging the aggregated experimental data for all 33 antibodies, it is found that there are peaks at sites 417, 444–456, and 484–490, with a peak at 484 being the largest peak (Figure S1), indicating that site E484 is the most common site that antibodies target with. In addition, there are smaller single peaks and peak clusters at a variety of other sites, such as 346, 472–476, and 493–504, showing that each antibody has a different epitope target RBD.

The average escape fractions of RBD variants towards these antibodies were used as biochemical properties to be correlated with computed molecular fields from each variant. The constructed MB-QSAR model also displayed quite well statistical parameters and good prediction power (Table 1, Figure 3d). The built MB-QSAR models were also employed to predict the escape fraction of circulating SARS-CoV-2 variants towards antibodies, which showed that the variants carrying the E484K mutation can significantly reduce the neutralization from polyclonal antibodies. The Omicron variants were predicted to display strong escape capacity from polyclonal antibody neutralization, mainly from the contribution of mutations such as E484A, Q493R, and N501Y (Table S5). These predicted results are consistent with prior studies about the neutralization of SARS-CoV-2 lineage with convalescent plasma and antibodies [46,55,56,57,58].

### 3.4. Molecular Interaction Diagram View of Variants

**Figure 5:**
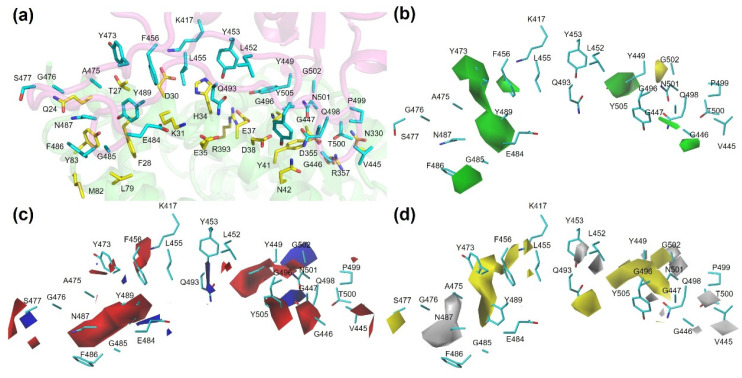
MB-QSAR/CoMSIA contour maps for the RBD-hACE2 binding interface. (**a**) The binding interface between wild-type RBD (cyan sticks) and hACE2 (yellow sticks). (**b**–**d**) Molecular field contour maps derived from the MB-QSAR/CoMSIA model, mapped onto the wild-type RBD structure. The contours indicate regions where specific molecular properties are predicted to increase (favored) or decrease (disfavored) the binding affinity (relative p*K*_D_) of RBD variants to hACE2. Steric field (**b**): Green contours (80% contribution level) indicate regions where increased steric bulk is favorable for binding. Yellow contours (20% level) indicate regions where decreased steric bulk is favorable. Electrostatic field (**c**): Blue contours (80% level) indicate regions where a positive charge is favorable for binding. Red contours (20% level) indicate regions where a negative charge is favorable. Hydrophobic field (**d**): Yellow contours (80% level) indicate regions where increased hydrophobicity is favorable for binding. Gray contours (20% level) indicate regions where increased hydrophilicity is favorable.

Here, we derived the 3D coefficient contour maps from CoMFA models, which can show the structural impacts on the binding of RBD variants to hACE2 and antibodies, thus providing a view of how the mutation affects the interactions between RBD and hACE2 as well as antibodies (Figure 5). The contours were mapped on the structure of wild-type RBD. The steric, electrostatic, and hydrophobic fields are represented by green and yellow, red and blue, as well as yellow and gray contours, respectively. The green and yellow areas in the steric field represent the favor or disfavor of steric bulk, which indicates that the bulky substituents in the green or yellow areas are favorable or unfavorable for the binding, respectively. In the electrostatic field, positively or negatively charged substituents in the blue or red areas are favored for binding, respectively. Hydrophobic or hydrophilic substituents in the yellow or gray areas of the hydrophobic field are favored for binding, respectively.

It has been shown that mutations that enhance affinity are notable at RBD sites L452, S477, E484, Q493, Q498, and N501. The blue contour found near S477 and E484 in the electrostatic field indicated that mutation of E484 to positively charged residues, such as S477K, as well as E484K and E484R, can enhance the binding to hACE2. In the hydrophobic field, the yellow contour near S477, N501, and Q493 showed that mutations of these sites to hydrophobic residues such as S477W, N501Y, N501F, and N501V, as well as Q493M and Q493Y are favorable for the binding of hACE2, while the gray contour found near L452 indicated that the mutation of L452 to hydrophilic residues such as L452R and L452Q enhance the binding to hACE2. In the steric field and hydrophobic, green and yellow contours were found near sites F456, Y473, Y489, and Y505, suggesting that the steric as well as hydrophobic effects of these residues are important for the binding of hACE2. Yellow contours found near G502 indicated that steric interaction is not favorable for this site. There were green contours found near F456, G446, Y473, G485, Y479, and Y505 in the steric field and gray contours near V445, L452, and G476 in the hydrophobic field. The mutations of these residues to the corresponding field did not show improved hACE2 binding affinity; instead, these mutations showed a slight decrease in the binding affinity, suggesting that these mutations might be tolerated during virus evolution. In addition, the red contours were found near K419, Y449, N487, Q498, and Y505 in the electrostatic field. The mutation of these residues to negatively charged residues reduced the binding affinity of RBD to hACE2. It was interesting to find that the interaction partners for the above residues found on hACE2, such as D30 for K419, D38 for Y449, N27 and Y83 for N487, N42 for Q498, and E37 for Y505, are mostly negatively charged or polar residues. Thus, these red contours can be explained.

**Figure 6:**
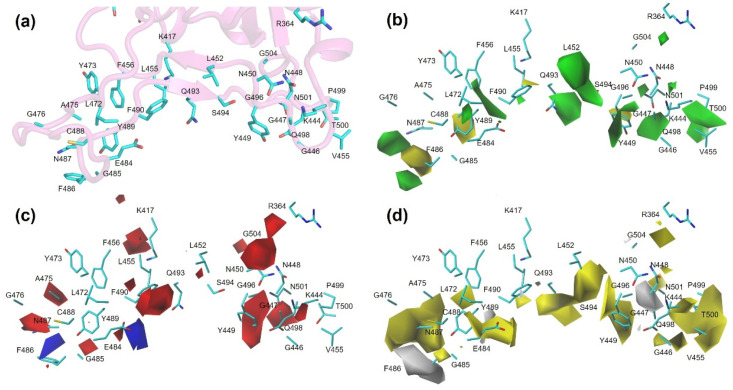
MB-QSAR/CoMSIA contour maps for the RBD-antibody binding interface. (**a**) The binding interface of wild-type RBD (cyan sticks) to a representative antibody. (**b**–**d**) Molecular field contour maps derived from the MB-QSAR/CoMSIA model, mapped onto the wild-type RBD structure. The contours indicate regions where specific molecular properties are predicted to increase the antibody escape fraction of RBD variants. Steric field (**b**): Green contours (80% contribution level) indicate regions where increased steric bulk favors antibody escape. Yellow contours (20% level) indicate regions where decreased steric bulk favors escape. Electrostatic field (**c**): Blue contours (80% level) indicate regions where a positive charge favors antibody escape. Red contours (20% level) indicate regions where a negative charge favors escape. Hydrophobic field (**d**): Yellow contours (80% level) indicate regions where increased hydrophobicity favors antibody escape. Gray contours (20% level) indicate regions where increased hydrophilicity favors escape.

For the MB-QSAR model of RBD variants to antibodies (Figure 6), we also found a strong correlation between the fields near RBD residues and the effect of mutations of these residues. For example, in the electrostatic field, blue contours were found near residue E484, N487, and F486; red contours were found near R346, K417, Y449, A475, F486, N487, and F490. This is consistent with the fact that the mutation of E484, N487, and F486 to the positively charged residue and the mutation of R346, K417, Y449, A475, F486, N487, and F490 to negatively charged residues can increase the escape fraction of RBD.

## 4. Discussion

The persistent evolution of SARS-CoV-2, characterized by the successive emergence of variants with augmented transmissibility and immune-evasive properties, highlights an urgent requirement for predictive methodologies capable of rapidly evaluating the phenotypic impacts of mutations. In this study, we have adapted and implemented a Mutation-dependent Biomacromolecular Quantitative Structure–Activity Relationship (MB-QSAR) framework to quantitatively model two critical biochemical phenotypes of SARS-CoV-2 receptor-binding domain (RBD) variants: their binding affinity to the human angiotensin-converting enzyme 2 (hACE2) receptor and their capacity to evade neutralization by a diverse panel of antibodies. Our models exhibit robust predictive accuracy, demonstrate generalizability to complex multi-mutant variants, and, importantly, yield interpretable structural rationales for their predictions.

A principal advantage of the MB-QSAR approach is its high predictive accuracy, achieving correlation coefficients (r^2^) exceeding 0.8 for hACE2 binding affinity and 0.7 for antibody escape. This performance is attained through the use of molecular field descriptors—a paradigm well-established in small-molecule drug design but less commonly applied to the systematic analysis of protein variants. Beyond its predictive performance, the MB-QSAR framework offers a theoretically grounded approach to modeling mutational effects. The method is rooted in the premise that mutations exert their phenotypic influence by altering the local physicochemical fields—steric, electrostatic, and hydrophobic—at the protein-protein interface. By representing variants through molecular field descriptors, MB-QSAR directly quantifies these biophysical perturbations, in contrast to sequence-based or embedding-based deep learning models that may capture complex patterns but often lack direct physicochemical interpretability. This makes MB-QSAR particularly suitable for analyzing a congeneric series of protein variants, as it explicitly encodes the fundamental forces driving molecular recognition. The resulting models are not only predictive but also provide visually interpretable contour maps that offer mechanistic insights into how specific field changes (e.g., introducing positive charge at E484) modulate biological activity, thereby bridging the gap between prediction and understanding.

Our results robustly validate the initial hypotheses. First, the high correlation coefficients and low prediction errors for both single- and multi-mutant variants confirm that molecular field perturbations induced by mutations can quantitatively capture their effects on protein–protein interaction. The model’s successful prediction of binding affinities for in vitro-evolved variants and circulating Omicron lineages (Figure 4) is particularly significant. This demonstrates that the model, trained primarily on single-point mutations, has effectively learned the fundamental physicochemical principles governing the RBD-hACE2 interface, enabling extrapolation to combinatorial mutational effects—a critical capability for forecasting the properties of emerging, complex variants.

Second, the model yields direct, visually interpretable mechanistic insights that corroborate and extend prior structural and functional studies. The derived contour maps (Figure 4 and Figure 5) provide a three-dimensional guide to mutational tolerance and enhancement within the RBD. For example, the presence of a blue contour (favoring positive charge) near E484 and a yellow contour (favoring hydrophobicity) near N501 offers a field-based explanation for the documented fitness advantages of E484K and N501Y mutations present in Variants of Concern (VOCs) [28,29,33]. These mutations are predicted to enhance electrostatic complementarity and hydrophobic packing, respectively, with hACE2, aligning with existing crystallographic and biophysical data [16]. Similarly, for antibody escape, the model accurately identifies E484 as a critical residue, with a blue contour indicating that substitutions with basic residues (E484K/R) efficiently disrupt antibody binding, consistent with its recognition as a recurrent escape hotspot across multiple antibody classes [25,46,55,58].

The differential field contributions between the RBD-hACE2 and RBD-antibody models are also informative. The greater contribution of steric fields to hACE2 binding suggests a tightly defined, shape-complementary interface, as observed in the complex structure [16]. In contrast, the predominance of electrostatic fields in antibody escape underscores the significance of specific charge-charge interactions in paratope recognition. This implies that viral evolution for immune evasion may frequently involve charge-reversing mutations that disrupt these key electrostatic interactions—a strategy evident in Omicron subvariants (e.g., E484A, Q493R).

The implications of this work extend beyond SARS-CoV-2. The MB-QSAR framework establishes a generalizable methodology for the quantitative analysis of mutational effects on any biomacromolecular interaction for which structural and phenotypic data exist. This has profound relevance for pandemic preparedness. By enabling the rapid in silico prioritization of high-risk mutations from genomic surveillance data, our approach can accelerate the risk assessment of novel pathogens or variants, thereby informing public health responses and guiding the preemptive development of countermeasures, such as broad-spectrum vaccines and therapeutic antibodies targeting resilient, mutationally constrained epitopes.

Future research directions are promising. First, while the current model focuses on the RBD, the MB-QSAR methodology can be extended to other viral proteins critical for replication or pathogenicity, or to host factors, to construct a more comprehensive model of viral fitness. Second, integrating MB-QSAR predictions with epidemiological data and models of viral transmission could help identify mutations with not only biochemical consequences but also high potential for global dissemination. Finally, the interpretable contour maps can be directly leveraged for the rational design of novel therapeutic antibodies and vaccine immunogens, focusing engineering efforts on residues where mutations are predicted to be most deleterious to viral fitness and thus less likely to emerge.

To better situate our MB-QSAR approach within the current ecosystem of computational methods, it is instructive to compare its performance and characteristics against established techniques, namely Molecular Dynamics (MD) and Machine Learning (ML). MD simulations, such as those used by Laurini et al. [37] and Chen et al. [34], provide high-resolution, dynamic insights and can achieve high accuracy (reported r^2^ ~0.7–0.9 for binding affinity prediction) but at a prohibitive computational cost—often requiring thousands of CPU/GPU hours per variant. This makes MD unsuitable for the high-throughput screening of thousands of mutations. In contrast, modern ML models, including deep learning frameworks like UniBind [39], offer exceptional speed and can directly learn from large DMS datasets. These models have demonstrated strong performance (e.g., UniBind reported r^2^ > 0.8 for hACE2 binding) but often function as “black boxes,” providing limited mechanistic insight into why a mutation has a specific effect.

The MB-QSAR framework occupies a strategic middle ground. In terms of accuracy, our models achieve r^2^ values exceeding 0.8 for hACE2 binding and 0.7 for antibody escape, which is comparable to the upper end of MD and ML performance. In terms of efficiency, while not as instantaneous as some ML inference, MB-QSAR is vastly more efficient than MD, enabling the rapid prediction of thousands of variants on standard computing hardware. Its principal advantage, however, lies in its unique blend of accuracy and interpretability. Unlike black-box ML models, MB-QSAR directly outputs 3D molecular interaction fields (Figure 5 and Figure 6) that offer a physicochemical rationale for its predictions, identifying whether steric, electrostatic, or hydrophobic effects drive a phenotypic change. This positions MB-QSAR not just as a predictive tool, but as a hypothesis-generating engine for understanding the structural basis of viral evolution and for guiding the rational design of interventions aimed at resilient epitopes.

It is also important to point out that, the applicability domain of our MB-QSAR models is primarily defined by the structural and chemical space of the training data. The models are most reliable for predicting the effects of single-point mutations at the 29 (for hACE2) and 35 (for antibodies) interfacial residues included in our training set. Predictions for multiple mutants, as shown for circulating variants, represent an extrapolation. The model’s accuracy in this regime (as validated in Figure 4) suggests it has learned generalizable physicochemical principles, but performance may decrease for combinations of mutations that introduce steric clashes or long-range electrostatic effects not represented in the training data.

While the MB-QSAR framework demonstrates strong predictive performance and valuable interpretability, several limitations should be noted. First, it is important to acknowledge its inherent limitation in explicitly modeling epistatic interactions—non-additive effects where the impact of one mutation depends on the presence of others. The current model primarily captures the additive, first-order effects of individual mutations on the molecular field landscape of the RBD. The observed predictive power for multi-mutant variants (Figure 4) suggests that for many mutations in the RBD-hACE2 and RBD-antibody interfaces, the effects are approximately additive or that the dominant effect of key mutations (e.g., E484K, N501Y) overshadows subtler epistatic interactions. This is consistent with some empirical findings in the literature for this system. However, we anticipate that the reliability of predictions may decrease for variants with highly complex mutation patterns where strong, long-range epistasis is known to play a decisive role. Future iterations of the MB-QSAR framework could be enhanced by incorporating explicit descriptors for pairwise or higher-order mutational interactions, potentially derived from co-variance analysis in sequence data or targeted molecular dynamics simulations, to more comprehensively address this challenge.

Second, an important consideration for our model is its treatment of electrostatic fields under a single, standard protonation state. As highlighted by insightful studies on the pH-dependence of spike protein behavior [59,60,61], electrostatic interactions are pivotal for binding and can be sensitive to environmental pH. For instance, pH shifts in different cellular compartments (e.g., endosomes) or microenvironments could alter the protonation states of key residues like histidines, aspartates, and glutamates, thereby modulating the electrostatic landscape and binding affinity. Our current MB-QSAR models, trained on structures generated at a standard protonation state, inherently average over these potential effects. While the strong predictive performance suggests that the dominant electrostatic features at physiological pH are captured, we acknowledge that the model may not fully account for binding mechanisms that are critically dependent on specific pH conditions. Future work could explore the construction of context-specific MB-QSAR models by explicitly modeling different protonation states or by incorporating pH as an explicit variable in the descriptor set, which would be invaluable for understanding viral entry mechanisms in more physiologically diverse scenarios.

Finally, the current model is specific to the RBD and its interactions. Its predictive power for mutations in other regions of the spike protein (e.g., the N-terminal domain) or for other viral proteins would require the development of new, tailored models.

Addressing these limitations—through the use of ensemble docking, incorporating explicit terms for epistasis, modeling multiple protonation states, and expanding the scope to other viral proteins—represents an exciting direction for future work that will further enhance the framework’s utility in forecasting viral evolution.

## 5. Conclusions

In conclusion, we have demonstrated that the MB-QSAR framework is a powerful and versatile tool for forecasting viral evolution. By delivering accurate, interpretable, and generalizable predictions of key phenotypic outcomes, it provides a valuable resource for the scientific community in the ongoing response to SARS-CoV-2 and in preparing for future viral threats.

## Figures and Tables

**Table 1:** Summary of statistical data for MB-QSAR analyses.

	RBD-hACE2		RBD-Antibodies	
	CoMFA	CoMSIA	CoMFA	CoMSIA
*ONC* ^a^	4	4	4	4
*q* ^2 b^	0.703	0.699	0.668	0.726
*SEE* ^c^	0.419	0.335	0.033	0.023
*r* ^2 d^	0.859	0.909	0.829	0.919
*F*-value ^e^	330.774	546.801	340.391	794.856
*r*^2^_pred_ ^f^	0.801	0.807	0.691	0.754
*SEP* ^g^	0.467	0.461	0.045	0.042
*CI* ^h^	0.430, 0.505	0.424, 0.498	0.041, 0.049	0.038, 0.045
Contributions ^i^				
S	0.532	0.243	0.414	0.194
E	0.468	0.427	0.586	0.501
H	/	0.329		0.305

^a ^*ONC*: optimal number of components; ^b ^*q*^2^: cross-validated squared correlation coefficient from leave-one-out (LOO); ^c ^*SEE*: standard error of estimate from non-cross-validation; ^d ^*r*^2^: square of the correlation coefficient of non-cross-validation; ^e ^*F*-value: *F*-test value; ^f ^*r*^2^_pred_: square of the correlation coefficient calculated from the test set; ^g^ *SEP*: Standard Error of Prediction; ^h ^*CI*: SEP 95% Confidence Interval; ^i^ Field contributions: S = steric field, E = electrostatic field, H = hydrophobic field.

## Data Availability

All the data are included in the Supplementary Materials.

## Supplementary Materials

The following supporting information can be downloaded at: https://www.mdpi.com/article/10.3390/biom15111538/s1, Figure S1: The distribution of binding affinity and escape fraction in training set and test set in MB-QSAR models; Figure S2: The escape map generated by aggregating 33 neutralizing antibodies targeting the SARS-CoV-2 RBD; Table S1: The experimental and predicted p*K*D values of RBD variants by MB-QSAR models; Table S2: SARS-COV-2 antibodies used in this study; Table S3: The experimental and predicted Escape fraction values of RBD variants to antibodies combination by MB-QSAR models; Table S4: The experimental and predicted p*K*D values of RBD variants with multiple mutations by MB-QSAR models; Table S5: the relative p*K*D of SARS-CoV-2 Omicron variants bound to hACE2.

## Abbreviations

The following abbreviations are used in this manuscript:

SARS-CoV-2	Severe Acute Respiratory Syndrome Coronavirus 2
RBD	the receptor-binding domain
MB-QSAR	Biomacromolecular Quantitative Structure–Activity Relationship
ACE2	Angiotensin-converting enzyme 2
hACE2	human ACE2 receptor
DMS	deep mutational scanning
COVID-19	Corona Virus Disease 2019
VOC	Variants of Concern
MD	molecular dynamics
ML	machine learning
CoMFA	Comparative Molecular Field Analysis
CoMSIA	Comparative Molecular Similarity Indices Analysis
PLS	partial least squares
LOO	leave-one-out
ONC	the optimal number of components
