# Supplementary material for: Modeling the Mutational Effects on Biochemical Phenotypes of SARS-CoV-2 Using Molecular Fields
Source: Biomolecules. 2025 Oct 31;15(11):1538. doi: 10.3390/biom15111538 (PMC12649838; doi:10.3390/biom15111538)
Supplement: Supplementary file 1 [file biomolecules-15-01538-s001.zip › biomolecules-3944836-supplementary.pdf]

Supporting Information

Modeling the mutational effects on biochemical phenotypes of SARS-CoV-2 using molecular fields

Baifan Wang*, Zhen Xi*

State Key Laboratory of Elemento-Organic Chemistry and Department of Chemical Biology, National Pesticide Engineering Research Center (Tianjin), Nankai University, 94 Weijin Road, Tianjin, 300071, China.

* Corresponding authors:

Dr. Baifan Wang

Email: Baifan_wang@outlook.com

Prof. Zhen Xi

E-mail: zhenxi@nankai.edu.cn (Z. Xi)

Tel: +86 022-23504782, Fax: +86 022-23504782.

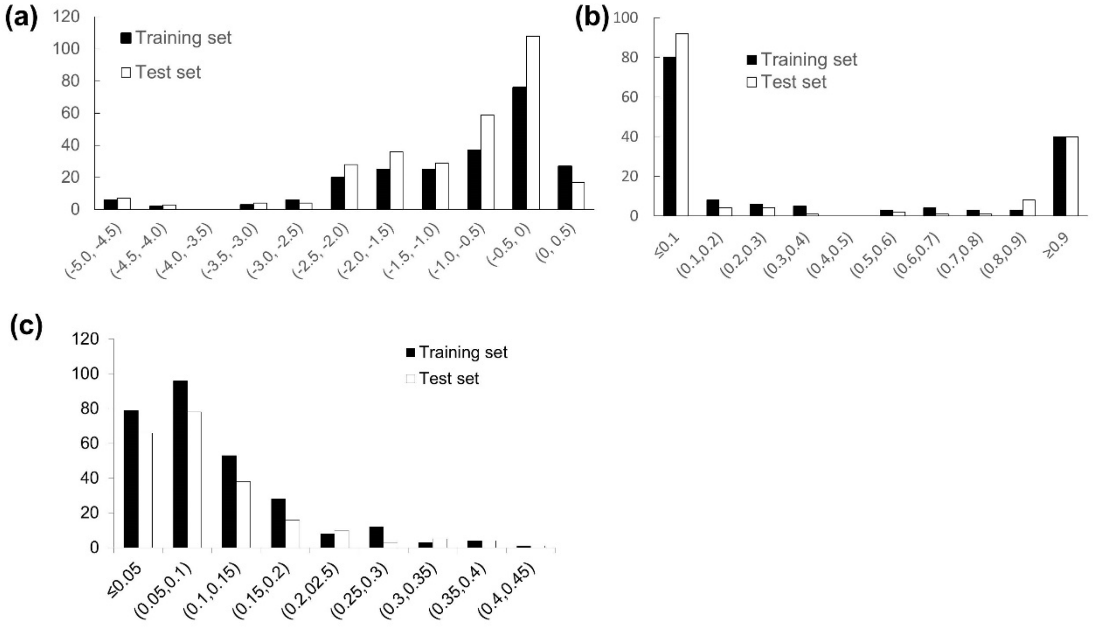

Figure S1. The distribution of binding affinity and escape fraction in training set and test set in MB-QSAR models. a: RBD-hACE2, b: RBD- LY-CoV016, c: RBD-antibody combinations.

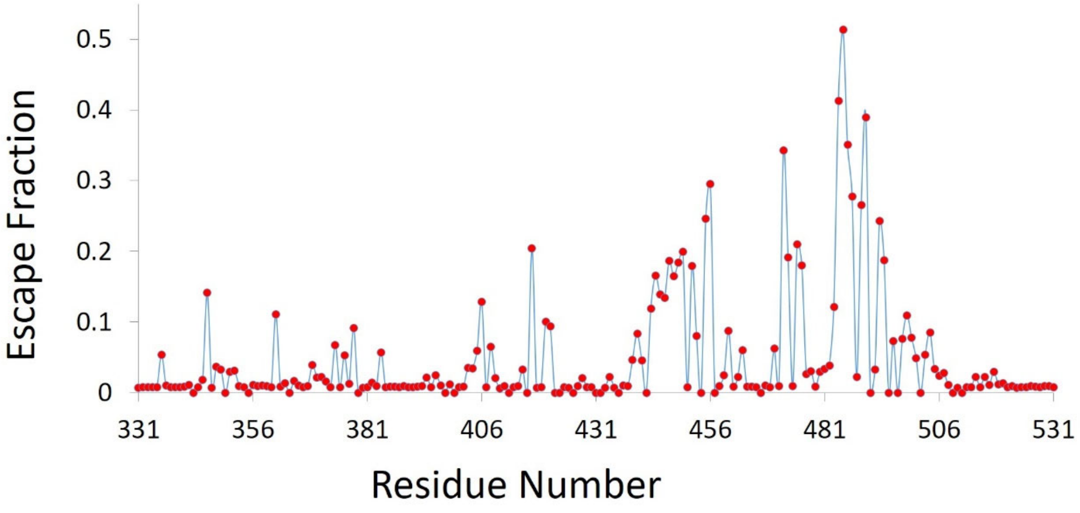

Figure S2. The escape map generated by aggregating 33 neutralizing antibodies targeting the SARS-CoV-2 RBD. The line shows the extent of escape mediated by mutations at each site, as estimated by averaging the data for all the individual antibodies.

Table S1. The experimental and predicted pK_D values of RBD variants by MB-QSAR models.

Training Set				Test set			
Mutations	Relative pK_D[a] (Exp)	Relative pK_D (CoMFA)	Relative pK_D (CoSIA)	Mutations	Relative pK_D[a] (Exp)	Relative pK_D (CoMFA)	Relative pK_D (CoSIA)
A475E	-0.480	-0.796	-0.739	Wild-type	0.000	-0.204	-0.282
A475I	-1.270	-1.127	-0.891	A475C	-0.660	-0.672	-0.912
A475L	-0.860	-1.326	-0.746	A475F	-1.380	-1.491	-1.626
A475R	-0.100	-0.852	-0.528	A475G	-0.130	-0.723	-0.869
A475S	-0.140	-0.828	-0.591	A475H	-1.980	-1.125	-1.199
A475V	-2.260	-1.899	-1.863	A475K	-1.060	-1.556	-0.859
A475W	-1.690	-1.525	-1.583	A475M	-0.380	-0.963	-0.867
A475Y	-0.070	-0.116	-0.306	A475N	-1.670	-0.623	-0.768
E484A	-0.380	-0.540	-0.347	A475P	-1.620	-0.709	-0.638
E484D	-0.550	-0.542	-0.631	A475Q	-0.800	-0.486	-0.755
E484H	-0.710	-0.233	-0.574	A475T	-0.360	-0.806	-0.604
E484L	-0.080	-0.197	-0.179	E484C	-0.520	-0.050	-0.183
E484N	0.150	0.432	0.141	E484F	-1.430	-0.586	-0.893
E484R	0.050	0.042	0.024	E484G	-0.060	-0.104	-0.267
E484T	-1.510	-0.809	-1.353	E484I	-0.640	-0.078	-0.369
E484Y	-2.170	-1.726	-1.638	E484K	0.060	0.817	-0.164
F456A	-2.450	-2.024	-2.211	E484M	-0.260	-0.318	-0.553
F456E	-1.210	-1.414	-1.196	E484P	-0.280	-0.300	-0.268
F456I	-1.380	-1.000	-1.079	E484Q	0.030	-0.321	-0.391
F456K	-0.110	-1.198	-0.735	E484S	-0.020	-0.143	0.045
F456L	-2.420	-1.749	-1.854	E484V	-0.650	-0.222	-0.473
F456S	-1.650	-1.766	-1.854	E484W	-0.780	-0.614	-0.741
F456T	-0.610	-1.392	-1.089	F456C	-1.200	-1.655	-1.604
F456V	-0.340	-0.547	-0.683	F456G	-2.220	-1.735	-1.798
F486A	-1.200	-0.577	-0.732	F456H	-0.690	-1.034	-1.263
F486C	-1.690	-1.118	-1.365	F456M	-0.220	-1.223	-1.198
F486D	-0.380	-0.628	-0.533	F456N	-2.380	-1.340	-1.664
F486I	-0.530	-0.785	-0.735	F456P	-2.690	-1.644	-1.502
F486M	-0.720	-0.711	-0.833	F456Q	-2.070	-1.503	-1.802
F486Q	-0.380	-0.772	-0.393	F456R	-2.080	-0.704	-1.133
F486R	-0.230	-0.899	-0.634	F456W	-1.010	-0.967	-0.814
F486W	-0.260	-0.439	-0.458	F456Y	-1.500	-0.731	-0.838
G446A	-0.970	-0.309	-0.384	F486E	-1.630	-1.162	-1.036
G446C	-0.310	-0.127	-0.275	F486G	-0.770	-0.537	-0.800
G446F	-0.280	-0.115	-0.222	F486H	-0.420	-0.751	-0.709
G446L	-0.110	-0.172	-0.106	F486K	-0.620	-0.448	-0.543
G446N	-0.160	-0.112	-0.042	F486L	-0.470	-0.744	-0.747
G446R	-0.200	-0.402	-0.278	F486N	-0.820	-0.828	-0.892

G446S	-0.270	-0.269	-0.237	F486P	-0.180	-0.449	-0.793
G446V	-1.180	-0.860	-1.093	F486S	-0.630	-0.559	-0.665
G447A	-1.760	-1.367	-1.572	F486T	-0.710	-0.615	-0.701
G447D	-1.680	-1.471	-1.730	F486V	-0.430	-0.607	-0.679
G447F	-1.870	-1.606	-1.582	F486Y	-0.340	-0.864	-0.818
G447H	-1.540	-1.174	-1.287	G446D	-0.200	-0.149	-0.222
G447L	-1.470	-1.133	-1.241	G446E	-0.120	-0.136	-0.286
G447N	-1.570	-1.399	-1.429	G446H	-0.280	-0.226	-0.190
G447R	-0.260	-0.741	-0.839	G446I	-0.260	-0.139	-0.765
G476A	-1.350	-1.111	-1.130	G446K	-0.090	-0.172	-0.225
G476E	-1.220	-0.959	-0.932	G446M	-0.230	-0.135	-0.136
G476F	-1.670	-1.026	-1.132	G446P	-0.260	-0.262	-0.148
G476M	-0.840	-1.096	-0.889	G446Q	-0.170	-0.169	-0.216
G476N	-0.020	-0.675	-0.563	G446T	-0.190	-0.323	-0.204
G476S	-1.960	-1.113	-1.617	G446W	-0.200	-0.159	-0.237
G476V	-0.570	-1.011	-0.784	G446Y	-0.340	-0.205	-0.198
G476W	-0.430	-0.363	-0.347	G447C	-1.780	-1.036	-1.158
G485E	-0.800	-0.653	-0.546	G447E	-1.630	-1.463	-1.874
G485F	-0.020	-0.182	-0.071	G447I	-1.550	-1.082	-1.602
G485K	-0.640	-0.369	-0.312	G447K	-1.540	-1.260	-1.408
G485L	-0.280	-0.389	-0.514	G447M	-1.530	-1.528	-1.635
G485M	-0.110	-0.191	-0.215	G447P	-1.810	-0.919	-0.734
G485Q	-0.570	-0.545	-0.474	G447Q	-1.590	-1.380	-1.209
G485W	-0.530	-0.822	-0.822	G447S	-1.240	-0.825	-0.987
G496A	-2.330	-1.981	-2.053	G447T	-1.400	-1.002	-1.014
G496E	-2.120	-1.621	-1.914	G447V	-1.520	-0.766	-1.409
G496F	-1.510	-1.404	-1.378	G447W	-1.740	-1.783	-2.230
G496I	-1.700	-1.252	-1.507	G447Y	-1.590	-1.599	-1.851
G496L	-0.350	-1.108	-0.907	G476C	-1.890	-0.770	-0.820
G496N	-1.630	-1.486	-1.371	G476D	-0.540	-0.879	-1.106
G496R	-1.050	-1.136	-1.156	G476H	-0.380	-0.936	-0.949
G496V	-2.800	-2.129	-2.286	G476I	-2.310	-0.985	-1.084
G502A	-4.160	-4.404	-4.932	G476K	-1.680	-1.122	-0.813
G502D	-4.800	-4.882	-5.530	G476L	-2.510	-0.907	-0.984
G502F	-4.160	-5.187	-4.746	G476Q	-1.120	-0.943	-0.902
G502K	-4.800	-5.161	-5.546	G476R	-1.180	-1.057	-0.834
G502L	-4.680	-4.829	-4.695	G476T	-0.890	-0.791	-0.583
G502Q	-2.700	-3.552	-3.471	G476Y	-0.980	-0.958	-1.110
G502S	-4.800	-4.446	-4.228	G485A	-0.190	-0.623	-0.726
G502T	-4.800	-4.470	-4.571	G485C	-0.590	-0.597	-0.631
G502V	-0.350	-0.560	-0.223	G485D	-0.580	-0.675	-0.666
K417A	-1.040	-1.185	-0.504	G485H	-0.450	-0.568	-0.586
K417D	-0.390	-0.663	-0.216	G485I	-0.660	-0.392	-0.400
K417M	-0.620	-0.711	-0.457	G485N	-0.430	-0.454	-0.629

K417Q	-0.170	-0.445	-0.173	G485P	-1.530	-0.683	-0.566
K417R	-0.460	-0.512	-0.237	G485R	-0.180	-0.254	-0.260
K417S	-0.320	-0.555	-0.203	G485S	-0.200	-0.622	-0.701
K417V	-0.310	-0.288	-0.160	G485T	-0.220	-0.664	-0.561
K417W	-0.080	-0.291	-0.082	G485V	-0.670	-0.563	-0.497
K417Y	-0.160	-0.354	0.020	G485Y	-0.700	-0.632	-0.569
L452D	0.090	0.095	0.253	G496C	-1.420	-0.940	-1.069
L452K	0.060	-0.310	-0.122	G496D	-2.210	-1.349	-1.439
L452M	0.070	-0.369	0.385	G496H	-1.520	-1.468	-1.856
L452Q	0.020	0.522	0.170	G496K	-1.650	-1.302	-0.930
L452R	0.030	-0.417	0.265	G496M	-1.620	-1.405	-1.536
L452S	-0.470	-0.672	-0.795	G496P	-2.100	-1.035	-0.731
L455A	-2.250	-1.638	-1.698	G496Q	-1.580	-1.207	-1.316
L455D	-0.190	-0.705	-0.698	G496S	-0.630	-0.894	-0.867
L455F	0.050	-0.802	-0.593	G496T	-1.460	-1.137	-1.004
L455M	-0.800	-0.926	-1.017	G496W	-1.630	-1.646	-1.943
L455N	-1.600	-0.738	-1.174	G496Y	-2.000	-1.519	-1.594
L455R	-0.730	-0.703	-0.670	G502C	-3.110	-3.974	-3.327
L455V	-1.500	-0.794	-0.961	G502E	-4.670	-4.235	-4.286
L455Y	-0.550	-0.685	-1.197	G502H	-4.470	-4.635	-4.796
N437A	-1.480	-0.797	-1.357	G502I	-4.660	-4.824	-5.208
N437D	-2.210	-0.940	-1.749	G502M	-4.800	-4.383	-4.198
N437I	-0.780	-0.975	-1.098	G502N	-4.380	-4.128	-3.477
N437K	-1.620	-1.055	-1.581	G502R	-4.800	-5.236	-4.442
N437M	-0.750	-0.927	-0.968	G502W	-4.800	-4.957	-5.669
N437Q	-1.230	-0.862	-1.288	G502Y	-4.800	-4.855	-5.537
N437T	-1.460	-0.886	-1.524	K417C	-0.420	-0.498	-0.343
N437V	-1.740	-1.651	-2.037	K417E	-0.750	-1.063	-0.218
N487A	-1.080	-1.125	-1.234	K417F	-0.130	-0.421	-0.104
N487D	-2.290	-2.050	-2.357	K417G	-0.510	-0.556	-0.209
N487F	-2.770	-2.187	-2.842	K417H	-0.170	-0.459	-0.114
N487I	-2.090	-1.541	-1.853	K417I	-0.340	-0.506	-0.390
N487Q	-2.300	-2.510	-2.227	K417L	-0.310	-0.563	-0.262
N487R	-2.470	-1.639	-2.598	K417N	-0.450	-0.832	-0.438
N487V	-0.060	-0.294	-0.231	K417P	-0.350	-0.500	-0.269
N501A	0.290	0.126	0.165	K417T	-0.260	-0.489	-0.359
N501F	0.000	-0.379	0.067	L452A	0.050	-0.376	-0.059
N501H	-0.080	-0.143	-0.143	L452C	-0.060	-0.439	0.015
N501I	0.000	-0.060	-0.004	L452E	0.020	-0.338	-0.084
N501M	0.150	-0.097	-0.109	L452F	-0.010	-0.503	-0.400
N501V	0.110	0.254	0.059	L452G	0.020	-0.376	0.199
N501W	-0.470	-0.742	-0.566	L452H	0.000	-0.550	-0.145
P499A	-1.100	-0.754	-1.065	L452I	0.000	-0.598	-0.362
P499E	-0.460	-0.412	-0.341	L452N	0.010	-0.380	0.113

P499I	-0.120	-0.441	-0.249	L452T	0.030	-0.440	0.097
P499K	-0.370	-0.494	-0.087	L452V	0.020	-0.450	-0.378
P499N	-0.150	-0.523	-0.224	L452W	-0.060	-0.491	-0.577
P499T	-0.990	-0.495	-0.523	L452Y	-0.070	-0.423	-0.350
P499W	-0.360	-0.372	-0.337	L455C	-0.710	-0.729	-0.756
P499Y	0.130	-0.001	-0.285	L455E	-2.050	-1.589	-0.764
Q493A	-1.570	-0.982	-1.160	L455G	-0.540	-0.659	-0.938
Q493D	-0.560	-0.883	-0.708	L455H	-0.760	-0.841	-0.961
Q493E	0.060	-0.191	-0.057	L455I	-0.010	-0.651	-0.742
Q493F	0.050	0.006	-0.151	L455K	-1.990	-0.497	-0.622
Q493L	0.180	-0.402	-0.169	L455P	-0.950	-0.719	-0.783
Q493M	-0.210	-0.383	-0.778	L455Q	-0.630	-0.933	-0.884
Q493N	-0.090	0.168	-0.066	L455S	-0.780	-0.661	-1.108
Q493R	-0.050	-0.255	-0.439	L455T	-0.780	-0.653	-1.018
Q498A	-1.270	-0.904	-0.666	L455W	-0.270	-0.860	-0.698
Q498D	0.150	-0.251	0.078	N437C	-0.420	-0.796	-1.222
Q498F	0.300	-0.205	0.291	N437E	-0.960	-0.933	-1.336
Q498H	-1.480	-0.457	-0.842	N437G	-0.560	-0.673	-1.034
Q498I	-0.510	-0.477	-0.470	N437H	-1.890	-0.898	-1.225
Q498M	-0.060	-0.341	0.121	N437P	-0.360	-0.778	-1.307
Q498R	-0.670	-0.232	-0.587	N437R	-0.750	-0.871	-1.086
Q498T	-0.130	-0.672	-0.432	N437S	-0.510	-0.795	-1.041
S375A	-0.500	-0.431	-0.393	N487C	-2.410	-1.664	-2.242
S375C	-0.550	-0.310	-0.342	N487E	-2.060	-1.653	-2.502
S375F	-0.690	-0.452	-0.455	N487G	-1.590	-1.611	-1.854
S375I	-0.130	-0.311	-0.113	N487H	-1.870	-1.667	-1.816
S375N	-0.370	-0.562	-0.287	N487K	-2.300	-2.001	-2.006
S375R	-0.060	-0.485	-0.277	N487L	-2.200	-1.184	-2.359
S375T	-0.550	-0.459	-0.412	N487M	-2.120	-1.701	-2.239
S375V	-0.070	-0.364	-0.284	N487P	-2.640	-1.693	-2.305
S477A	0.090	0.324	0.064	N487S	-1.510	-1.604	-1.958
S477D	-0.050	0.043	0.062	N487T	-1.930	-1.691	-2.248
S477F	-0.060	-0.043	-0.161	N487W	-2.390	-1.764	-2.217
S477I	0.030	-0.179	-0.067	N487Y	-2.300	-2.169	-2.596
S477K	0.060	0.121	-0.021	N501C	-0.580	-0.138	-0.286
S477N	-0.010	-0.073	-0.051	N501D	-2.420	-1.336	-0.761
S477Q	-0.050	0.184	-0.045	N501E	-0.790	-0.903	-0.429
S477V	-0.560	-0.760	-1.068	N501G	-0.940	-0.253	-0.502
T500A	-1.990	-1.490	-1.415	N501Q	-0.060	-0.495	-0.413
T500D	-2.300	-1.187	-1.496	N501S	-0.130	-0.108	-0.395
T500I	-1.520	-1.242	-1.318	N501T	0.100	-0.242	-0.259
T500L	-0.750	-1.073	-1.198	N501Y	0.240	0.527	0.068
T500M	-1.120	-0.937	-0.922	P499C	-1.070	-0.546	-0.366
T500R	-0.320	-0.782	-0.727	P499D	-0.920	-0.741	-0.375

T500S	-0.430	-0.624	-0.846	P499F	-0.410	-0.469	-0.432
T500W	-0.040	-0.267	-0.139	P499G	-1.020	-0.805	-0.508
V445A	-0.180	0.278	0.201	P499H	-0.470	-0.550	-0.409
V445E	-0.100	0.133	-0.039	P499L	-0.360	-0.454	-0.647
V445F	-0.010	0.082	-0.125	P499M	-0.460	-0.602	-0.428
V445I	-0.060	0.005	0.280	P499Q	-0.590	-0.580	-0.516
V445N	-0.010	-0.030	0.201	P499R	-0.140	-0.374	-0.446
V445R	-0.100	-0.333	-0.099	P499S	-0.230	-0.615	-0.352
V445S	-1.570	-1.758	-1.573	P499V	-0.370	-0.589	-0.406
Y449E	-1.080	-1.026	-1.062	Q493C	-0.030	-0.098	-0.413
Y449F	-1.510	-1.559	-1.331	Q493G	0.010	-0.008	-0.347
Y449L	-1.220	-1.478	-1.239	Q493H	-0.010	-0.143	-0.383
Y449Q	-0.690	-0.858	-0.677	Q493I	-0.080	-0.266	-0.318
Y449R	-1.250	-1.333	-1.335	Q493K	0.050	-0.061	-0.346
Y449S	-1.360	-1.362	-1.325	Q493P	-0.720	0.059	-0.217
Y449V	-1.430	-1.535	-1.187	Q493S	-0.180	0.085	-0.492
Y449W	-1.180	-1.018	-1.142	Q493T	-0.080	0.092	-0.624
Y453E	0.250	-0.477	-0.522	Q493V	0.050	-0.021	-0.237
Y453F	-0.070	-0.454	-0.377	Q493W	-0.140	-0.352	-0.312
Y453H	0.080	0.280	0.117	Q493Y	0.120	-0.144	-0.219
Y453K	-0.170	-0.314	-0.390	Q498C	-1.410	-0.399	-0.612
Y453L	-0.820	-0.336	-0.640	Q498E	-0.980	-0.994	-1.216
Y453N	-0.300	-0.057	-0.087	Q498G	-0.610	-0.230	-0.446
Y453R	-0.550	-0.234	-0.346	Q498L	-1.580	-0.553	-0.556
Y453T	-2.180	-2.095	-1.796	Q498N	-0.500	-0.492	-0.336
Y473A	-2.720	-2.507	-2.331	Q498S	-0.170	-0.235	-0.487
Y473D	-2.740	-2.205	-2.197	Q498V	-1.240	-0.406	-0.575
Y473G	-0.160	-1.140	-0.928	Q498W	0.070	-0.530	-0.335
Y473H	-0.650	-1.132	-0.961	Q498Y	0.160	-0.148	-0.014
Y473L	-0.660	-1.060	-1.061	S375D	-0.040	-0.216	-0.056
Y473M	-1.950	-1.586	-1.947	S375E	-0.110	-0.204	-0.262
Y473Q	-2.460	-3.168	-2.513	S375G	-0.200	-0.689	-0.370
Y489A	-3.370	-3.300	-2.806	S375H	-0.130	-0.232	-0.355
Y489D	-0.950	-1.189	-1.102	S375K	-0.230	-0.497	-0.576
Y489F	-0.660	-1.272	-1.019	S375L	-0.600	-0.400	-0.280
Y489H	-2.220	-2.950	-2.344	S375M	-0.440	-0.402	-0.506
Y489I	-1.980	-2.058	-2.045	S375P	-0.400	-0.662	-0.441
Y489M	-2.200	-2.275	-2.338	S375Q	-0.030	-0.331	-0.364
Y489Q	-4.800	-3.402	-3.368	S375W	-0.530	-0.377	-0.308
Y489S	-2.430	-2.842	-2.469	S375Y	-0.290	-0.264	-0.214
Y505A	-3.070	-2.813	-2.829	S477C	-0.440	-0.054	-0.194
Y505D	-0.150	-0.965	-1.090	S477E	-0.040	-0.012	-0.047
Y505F	-2.710	-2.686	-2.474	S477G	-0.060	-0.501	-0.267
Y505K	-2.320	-2.241	-2.201	S477H	-0.020	-0.063	0.010

Y505M	-2.370	-2.795	-2.819
Y505S	-3.460	-2.833	-2.825
Y505T	0.020	-0.595	-0.602
Y508F	-0.570	-0.472	-0.571
Y508I	-1.230	-0.999	-1.135
Y508K	-0.380	-0.462	-0.441
Y508Q	-0.400	-0.376	-0.512
Y508S	-0.250	-0.382	-0.625
Y508V			
S477L	-0.020	-0.072	0.053
S477M	-0.070	-0.061	-0.042
S477P	0.060	-0.405	-0.273
S477R	-0.030	-0.248	-0.012
S477T	-0.010	-0.373	-0.208
S477W	0.020	0.024	0.048
S477Y	-0.020	0.041	0.090
T500C	-1.600	-0.828	-0.974
T500E	-1.140	-1.567	-1.264
T500F	-0.980	-0.671	-1.262
T500G	-0.930	-0.662	-0.849
T500H	-0.880	-0.675	-1.345
T500K	-0.800	-0.770	-0.988
T500N	-1.200	-0.943	-1.109
T500P	-1.120	-0.830	-1.033
T500Q	-0.440	-1.191	-1.000
T500V	-1.890	-0.791	-0.958
T500Y	-0.970	-0.676	-1.156
V445C	-0.800	-0.332	-0.400
V445D	-0.160	0.113	-0.043
V445G	-0.290	-0.226	-0.027
V445H	-0.020	0.131	0.062
V445K	0.020	0.099	-0.010
V445L	-0.030	-0.112	-0.147
V445M	-0.140	0.049	-0.122
V445P	0.030	-0.323	-0.160
V445Q	-0.040	-0.067	0.148
V445T	-0.020	-0.464	-0.505
V445W	-0.030	0.130	-0.180
V445Y	-0.140	0.182	-0.224
Y449A	-1.300	-1.171	-1.169
Y449C	-1.730	-1.387	-1.207
Y449D	-1.550	-1.603	-1.247
Y449G	-1.280	-1.173	-1.253
Y449H	-1.220	-1.214	-1.039
Y449I	-1.440	-1.548	-1.383
Y449K	-0.980	-1.349	-1.083
Y449M	-1.370	-1.442	-1.276
Y449N	-1.220	-1.386	-1.194
Y449P	-1.410	-1.170	-1.240
Y449T	-1.160	-1.302	-1.190
Y453A	-0.270	-0.266	-0.493
Y453C	-0.260	-0.262	-0.517
Y453G	-0.920	-0.272	-0.442

Y453I	-0.530	-0.297	-0.347
Y453M	-0.050	-0.330	-0.475
Y453Q	-0.370	-0.506	-0.786
Y453S	-0.210	-0.277	-0.381
Y453V	-0.110	-0.284	-0.416
Y453W	-0.160	-0.343	-0.536
Y473C	-2.100	-1.799	-1.729
Y473E	-1.860	-1.934	-2.271
Y473F	-0.160	-0.956	-0.885
Y473I	-1.230	-1.341	-1.230
Y473N	-2.230	-1.539	-1.849
Y473S	-2.130	-1.921	-1.789
Y473T	-1.850	-1.821	-1.653
Y473V	-1.200	-1.754	-1.417
Y473W	-0.810	-0.936	-0.844
Y489C	-3.240	-3.375	-2.792
Y489G	-4.320	-3.127	-2.712
Y489L	-2.200	-1.833	-1.982
Y489N	-2.570	-2.408	-2.596
Y489P	-2.260	-2.775	-2.307
Y489T	-4.610	-3.252	-3.113
Y489V	-2.150	-2.897	-2.152
Y489W	-0.680	-1.368	-1.012
Y505C	-2.360	-2.822	-2.563
Y505E	-3.170	-2.635	-2.450
Y505H	-0.710	-1.478	-1.715
Y505I	-3.190	-2.389	-2.196
Y505L	-2.510	-1.807	-2.257
Y505N	-2.000	-2.502	-2.607
Y505Q	-2.290	-2.369	-2.734
Y505R	-2.470	-1.192	-1.996
Y505V	-2.460	-2.390	-2.442
Y508C	-0.330	-0.357	-0.644
Y508E	-1.140	-0.369	-0.323
Y508H	0.070	-0.598	-0.537
Y508L	-0.520	-0.570	-0.631
Y508M	-0.180	-0.675	-0.524
Y508N	-0.190	-0.423	-0.351
Y508R	-0.770	-0.752	-0.605
Y508T	-0.270	-0.397	-0.544
Y508W	-0.170	-0.648	-0.362

Table S2. SARS-COV-2 antibodies used in this study.

Class	Name
1	C105
	COV2-2165
	COV2-2196
	COV2-2832
	LY-CoV016
	REGN10933
	S2E12
	S2H14
2	C002
	C121
	C144
	COV2-2050
	COV2-2096
	COV2-2955
	COV2-2479
	LY-CoV555
	S2D106
	S2H13
	S2H58
	S2X16
	S2X58
3	C110
	C135
	COV2-2130
	COV2-2499
	REGN10987
	S2X227
4	COV2-2082
	COV2-2094
	COV2-2677
	S2H97
	S2X259
	S2X35

Table S3. The experimental and predicted Escape fraction values of RBD variants to antibodies combination by MB-QSAR models.

Training set				Test set			
Mutation	Escape fraction (EXP)	Escape fraction (CoMFA)	Escape fraction (CoMSIA)	Mutation	Escape fraction (EXP)	Escape fraction (CoMFA)	Escape fraction (CoMSIA)
A475C	0.033	0.089	0.071	Wild-type	0.000	0.098	0.074
A475D	0.184	0.090	0.126	A475E	0.150	0.068	0.092
A475G	0.023	0.084	0.080	A475F	0.114	0.081	0.084
A475I	0.084	0.084	0.072	A475K	0.132	0.101	0.050
A475M	0.061	0.067	0.071	A475L	0.035	0.083	0.067
A475Q	0.061	0.085	0.071	A475T	0.024	0.100	0.063
A475R	0.115	0.103	0.071	A475V	0.024	0.091	0.056
A475S	0.009	0.094	0.061	D405C	0.000	0.004	0.004
A475Y	0.090	0.079	0.088	D405H	0.000	0.038	0.007
D405A	0.279	0.015	0.008	D405I	0.000	-0.004	0.004
D405E	0.013	0.030	0.001	D405M	0.000	0.021	0.011
D405F	0.003	0.029	0.007	D405P	0.000	0.021	0.001
D405G	0.000	0.015	0.010	D405Q	0.000	0.024	0.002
D405K	0.052	0.011	0.031	D405R	0.031	0.008	0.027
D405L	0.001	0.045	0.014	D405T	0.000	0.005	0.011
D405N	0.058	0.055	0.048	D405W	0.000	0.032	0.005
D405S	0.000	0.003	0.010	D405Y	0.000	0.034	0.009
D405V	0.000	0.007	0.006	D420E	0.090	0.101	0.082
D420A	0.084	0.081	0.070	D420K	0.101	0.093	0.083
D420M	0.081	0.091	0.079	D420N	0.087	0.077	0.078
D420Q	0.096	0.092	0.079	E484C	0.300	0.395	0.359
D420R	0.100	0.103	0.088	E484D	0.329	0.243	0.255
D420S	0.043	0.078	0.059	E484F	0.208	0.398	0.381
E484A	0.372	0.394	0.350	E484H	0.412	0.378	0.356
E484I	0.395	0.378	0.394	E484M	0.399	0.310	0.305
E484K	0.259	0.421	0.413	E484N	0.399	0.327	0.294
E484L	0.385	0.373	0.367	E484P	0.356	0.409	0.358
E484V	0.396	0.396	0.383	E484Q	0.328	0.305	0.214
E484Y	0.413	0.406	0.409	E484R	0.343	0.417	0.415
F456H	0.123	0.094	0.123	E484W	0.344	0.392	0.371
F456I	0.136	0.122	0.154	F456C	0.179	0.129	0.176
F456Q	0.214	0.124	0.203	F456K	0.236	0.171	0.173
F456R	0.191	0.168	0.212	F456L	0.063	0.110	0.122
F456V	0.151	0.121	0.155	F456M	0.072	0.124	0.149
F456Y	0.091	0.082	0.088	F456T	0.214	0.129	0.187
F486D	0.277	0.251	0.241	F486A	0.218	0.285	0.273
F486G	0.282	0.303	0.292	F486C	0.217	0.265	0.256
F486H	0.149	0.152	0.182	F486E	0.277	0.243	0.257

F486K	0.309	0.331	0.317	F486I	0.228	0.181	0.222
F486L	0.161	0.183	0.211	F486N	0.210	0.261	0.286
F486Q	0.253	0.278	0.300	F486P	0.350	0.283	0.280
F486R	0.296	0.223	0.287	F486S	0.254	0.269	0.307
F486T	0.303	0.280	0.294	F486W	0.086	0.182	0.139
F486V	0.221	0.257	0.243	F490A	0.133	0.209	0.163
F486Y	0.065	0.099	0.081	F490H	0.057	0.125	0.144
F490D	0.318	0.334	0.286	F490L	0.199	0.164	0.148
F490E	0.294	0.309	0.268	F490P	0.256	0.218	0.228
F490G	0.180	0.218	0.171	F490S	0.126	0.207	0.180
F490I	0.175	0.200	0.143	F490Y	0.068	0.078	0.079
F490N	0.275	0.227	0.271	G446A	0.033	0.088	0.086
F490Q	0.261	0.262	0.233	G446D	0.079	0.088	0.089
F490T	0.189	0.218	0.174	G446F	0.077	0.090	0.099
F490V	0.161	0.195	0.147	G446H	0.095	0.088	0.094
F490W	0.089	0.089	0.085	G446L	0.104	0.091	0.095
G446C	0.081	0.087	0.091	G446M	0.070	0.097	0.094
G446E	0.106	0.096	0.101	G446P	0.134	0.094	0.095
G446I	0.102	0.093	0.100	G446Q	0.088	0.092	0.095
G446K	0.127	0.093	0.099	G446R	0.117	0.096	0.092
G446N	0.089	0.098	0.083	G446S	0.041	0.086	0.090
G446T	0.091	0.091	0.093	G446W	0.084	0.089	0.098
G446V	0.121	0.092	0.096	G446Y	0.080	0.094	0.097
G447A	0.022	0.084	0.068	G447E	0.157	0.081	0.079
G447C	0.049	0.086	0.087	G447F	0.137	0.073	0.102
G447D	0.186	0.093	0.154	G447L	0.151	0.085	0.089
G447H	0.096	0.082	0.106	G447M	0.083	0.071	0.082
G447I	0.086	0.079	0.073	G447P	0.150	0.084	0.058
G447K	0.066	0.074	0.062	G447Q	0.105	0.084	0.078
G447N	0.116	0.088	0.102	G447R	0.040	0.084	0.064
G447T	0.067	0.081	0.078	G447S	0.052	0.088	0.079
G447V	0.097	0.066	0.078	G476A	0.010	0.092	0.084
G447Y	0.108	0.073	0.123	G476N	0.103	0.096	0.077
G476D	0.109	0.092	0.066	G476R	0.145	0.148	0.126
G476H	0.054	0.098	0.075	G476S	0.015	0.098	0.079
G476K	0.137	0.160	0.138	G485E	0.104	0.111	0.094
G476Q	0.086	0.089	0.086	G485Q	0.061	0.089	0.085
G476T	0.107	0.098	0.087	G485R	0.126	0.055	0.070
G485A	0.046	0.078	0.076	G485T	0.123	0.075	0.066
G485C	0.068	0.084	0.069	G496A	0.007	0.080	0.057
G485D	0.167	0.122	0.119	G496E	0.073	0.069	0.024
G485H	0.093	0.080	0.079	G496F	0.022	0.050	0.038
G485K	0.098	0.054	0.071	G496H	0.037	0.053	0.020
G485M	0.060	0.076	0.078	G496I	0.045	0.060	0.019

G485N	0.101	0.096	0.091	G496K	0.021	0.066	0.036
G485S	0.041	0.077	0.064	G496L	0.045	0.069	0.028
G496C	0.027	0.077	0.054	G496M	0.041	0.069	0.041
G496D	0.055	0.074	0.047	G496Q	0.048	0.064	0.015
G496N	0.045	0.068	0.038	G496S	0.009	0.081	0.053
G496R	0.060	0.059	0.041	G496W	0.048	0.069	0.050
G496T	0.017	0.064	0.040	G504E	0.061	0.046	0.051
G496V	0.050	0.071	0.031	G504L	0.079	0.041	0.047
G496Y	0.024	0.044	0.039	G504M	0.073	0.046	0.062
G504A	0.032	0.069	0.067	G504P	0.064	0.063	0.073
G504C	0.076	0.052	0.062	G504Q	0.042	0.035	0.062
G504D	0.064	0.049	0.051	G504R	0.043	0.024	0.077
G504F	0.067	0.050	0.071	G504S	0.031	0.055	0.075
G504H	0.059	0.069	0.067	G504W	0.053	0.044	0.054
G504I	0.085	0.039	0.062	G504Y	0.055	0.048	0.075
G504K	0.071	0.035	0.082	I472A	0.059	0.089	0.067
G504N	0.045	0.030	0.040	I472H	0.065	0.085	0.085
G504T	0.058	0.054	0.075	I472L	0.010	0.091	0.096
G504V	0.077	0.056	0.059	I472M	0.034	0.095	0.096
I472C	0.080	0.087	0.069	I472Q	0.053	0.080	0.110
I472E	0.147	0.102	0.114	I472T	0.015	0.086	0.080
I472F	0.064	0.090	0.067	I472V	0.011	0.091	0.066
I472K	0.186	0.111	0.159	I472Y	0.079	0.107	0.099
I472N	0.073	0.083	0.073	K417G	0.134	0.092	0.140
I472P	0.017	0.086	0.055	K417I	0.103	0.094	0.125
I472S	0.100	0.085	0.082	K417M	0.075	0.104	0.133
I472W	0.214	0.173	0.199	K417Q	0.087	0.110	0.169
K417A	0.095	0.097	0.135	K417R	0.046	0.084	0.099
K417C	0.114	0.094	0.135	K417S	0.103	0.093	0.139
K417D	0.204	0.132	0.190	K417T	0.095	0.095	0.141
K417E	0.190	0.134	0.230	K417W	0.163	0.105	0.138
K417F	0.150	0.105	0.152	K417Y	0.147	0.102	0.146
K417H	0.120	0.105	0.153	K444C	0.111	0.142	0.133
K417L	0.102	0.095	0.135	K444E	0.149	0.173	0.165
K417N	0.100	0.092	0.145	K444G	0.128	0.141	0.127
K417V	0.111	0.095	0.134	K444L	0.157	0.138	0.146
K444A	0.133	0.142	0.135	K444M	0.129	0.133	0.130
K444D	0.146	0.168	0.162	K444N	0.089	0.148	0.145
K444F	0.141	0.136	0.149	K444T	0.103	0.142	0.132
K444H	0.107	0.140	0.135	K444W	0.157	0.129	0.149
K444I	0.153	0.144	0.149	K444Y	0.153	0.132	0.154
K444P	0.166	0.150	0.170	L452G	0.052	0.056	0.055
K444Q	0.125	0.143	0.131	L452H	0.068	0.083	0.075
K444R	0.053	0.081	0.067	L452M	0.011	0.103	0.094

K444S	0.100	0.139	0.125	L452Q	0.049	0.104	0.096
K444V	0.143	0.145	0.141	L452T	0.111	0.052	0.050
L452A	0.041	0.053	0.053	L452Y	0.096	0.104	0.085
L452C	0.039	0.054	0.043	L455A	0.166	0.120	0.131
L452D	0.107	0.113	0.089	L455I	0.008	0.098	0.081
L452E	0.140	0.159	0.098	L455M	0.023	0.096	0.104
L452F	0.089	0.098	0.085	L455T	0.063	0.116	0.130
L452I	0.010	0.068	0.039	L455W	0.142	0.118	0.100
L452K	0.148	0.070	0.132	L455Y	0.061	0.088	0.075
L452N	0.050	0.084	0.062	N440A	0.008	0.088	0.063
L452R	0.179	0.081	0.148	N440C	0.013	0.087	0.063
L452S	0.088	0.049	0.053	N440D	0.039	0.099	0.077
L452V	0.024	0.068	0.044	N440E	0.029	0.098	0.072
L452W	0.160	0.112	0.145	N440G	0.010	0.087	0.067
L455C	0.071	0.116	0.119	N440H	0.009	0.086	0.067
L455F	0.082	0.096	0.062	N440M	0.031	0.082	0.059
L455G	0.203	0.132	0.148	N440P	0.009	0.084	0.063
L455H	0.150	0.099	0.124	N440Q	0.014	0.082	0.068
L455N	0.113	0.090	0.118	N440R	0.016	0.068	0.059
L455P	0.113	0.121	0.121	N440S	0.008	0.090	0.066
L455R	0.227	0.194	0.209	N440T	0.022	0.083	0.063
L455S	0.157	0.122	0.143	N440W	0.000	0.083	0.060
N440I	0.067	0.079	0.057	N440Y	0.029	0.084	0.060
N440K	0.056	0.065	0.048	N448C	0.045	0.080	0.074
N440L	0.081	0.086	0.057	N448E	0.051	0.089	0.064
N448A	0.028	0.078	0.075	N448R	0.144	0.062	0.084
N448D	0.085	0.101	0.071	N450G	0.038	0.048	0.044
N448G	0.077	0.076	0.072	N450M	0.035	0.050	0.047
N448H	0.027	0.078	0.052	N450Q	0.033	0.050	0.051
N448K	0.134	0.074	0.108	N450R	0.009	0.013	0.016
N448P	0.165	0.078	0.108	N450W	0.041	0.072	0.045
N448Q	0.057	0.079	0.063	N450Y	0.043	0.040	0.038
N448T	0.051	0.080	0.068	N460C	0.045	0.053	0.051
N448V	0.103	0.076	0.100	N460D	0.010	0.049	0.079
N448Y	0.084	0.080	0.072	N460G	0.030	0.057	0.059
N450A	0.037	0.050	0.043	N460I	0.062	0.056	0.056
N450C	0.019	0.056	0.036	N460M	0.062	0.068	0.065
N450D	0.093	0.126	0.078	N460P	0.061	0.063	0.056
N450F	0.040	0.048	0.041	N460R	0.058	0.084	0.063
N450H	0.010	0.028	0.026	N460T	0.057	0.059	0.057
N450I	0.038	0.053	0.043	N460Y	0.084	0.067	0.073
N450K	0.012	0.026	0.013	N487D	0.176	0.109	0.062
N450L	0.035	0.055	0.041	N487G	0.088	0.149	0.151
N450S	0.034	0.052	0.040	N487L	0.144	0.118	0.178

N450V	0.033	0.058	0.039	N487S	0.123	0.151	0.145
N460A	0.058	0.054	0.057	N487Y	0.215	0.216	0.147
N460E	0.070	0.052	0.061	N501C	0.035	0.071	0.044
N460F	0.087	0.071	0.072	N501F	0.029	0.048	0.029
N460H	0.056	0.069	0.065	N501G	0.034	0.073	0.052
N460K	0.061	0.073	0.062	N501H	0.034	0.062	0.049
N460L	0.086	0.078	0.070	N501I	0.031	0.059	0.035
N460Q	0.052	0.059	0.057	N501L	0.037	0.073	0.039
N460S	0.049	0.055	0.049	N501P	0.031	0.072	0.036
N460V	0.052	0.056	0.055	N501Q	0.037	0.077	0.050
N487A	0.127	0.152	0.159	N501S	0.035	0.074	0.048
N487E	0.184	0.147	0.161	P499A	0.008	0.081	0.054
N487F	0.194	0.202	0.155	P499E	0.084	0.085	0.078
N487H	0.133	0.167	0.166	P499L	0.010	0.073	0.050
N487K	0.257	0.204	0.245	P499Q	0.047	0.080	0.053
N487M	0.128	0.169	0.180	P499S	0.043	0.081	0.035
N487Q	0.144	0.136	0.156	P499V	0.009	0.075	0.048
N487R	0.277	0.267	0.274	Q493A	0.073	0.077	0.098
N487T	0.237	0.154	0.184	Q493C	0.085	0.079	0.104
N501M	0.041	0.063	0.035	Q493E	0.060	0.053	0.071
N501R	0.048	0.036	0.030	Q493G	0.060	0.080	0.101
N501T	0.034	0.068	0.035	Q493H	0.020	0.114	0.119
N501V	0.039	0.070	0.031	Q493N	0.013	0.073	0.107
N501W	0.037	0.030	0.045	Q493V	0.095	0.084	0.100
N501Y	0.033	0.041	0.030	Q498A	0.009	0.044	0.053
P499C	0.054	0.077	0.044	Q498C	0.034	0.037	0.059
P499D	0.109	0.086	0.059	Q498E	0.046	0.082	0.069
P499G	0.072	0.083	0.056	Q498G	0.038	0.040	0.062
P499H	0.047	0.064	0.029	Q498H	0.033	0.061	0.057
P499M	0.016	0.062	0.034	Q498L	0.031	0.045	0.056
P499N	0.044	0.069	0.036	Q498M	0.041	0.057	0.057
P499R	0.077	0.063	0.054	Q498N	0.036	0.047	0.056
P499T	0.029	0.069	0.035	Q498R	0.053	0.062	0.055
P499Y	0.045	0.061	0.040	Q498T	0.012	0.043	0.039
Q493F	0.136	0.111	0.133	Q498W	0.076	0.071	0.048
Q493I	0.097	0.075	0.101	R346C	0.089	0.081	0.069
Q493L	0.094	0.093	0.097	R346E	0.142	0.136	0.083
Q493R	0.191	0.147	0.180	R346G	0.059	0.083	0.068
Q493W	0.135	0.126	0.126	R346I	0.055	0.088	0.071
Q493Y	0.135	0.114	0.132	R346M	0.076	0.082	0.070
Q498D	0.034	0.053	0.043	R346Q	0.063	0.097	0.069
Q498F	0.071	0.067	0.059	R346T	0.076	0.080	0.069
Q498I	0.049	0.039	0.035	R346W	0.062	0.078	0.071
Q498K	0.059	0.057	0.055	R346Y	0.073	0.087	0.066

Q498S	0.008	0.037	0.053	S494A	0.009	0.086	0.073
Q498V	0.012	0.042	0.050	S494M	0.087	0.091	0.082
Q498Y	0.059	0.061	0.071	S494R	0.187	0.085	0.101
R346A	0.061	0.081	0.063	S494V	0.065	0.088	0.092
R346D	0.139	0.122	0.110	S494W	0.125	0.089	0.096
R346F	0.066	0.079	0.070	T500E	0.047	0.064	0.039
R346H	0.044	0.076	0.057	T500G	0.021	0.065	0.037
R346K	0.054	0.017	0.041	T500L	0.033	0.045	0.029
R346L	0.093	0.086	0.073	T500M	0.036	0.031	0.031
R346N	0.059	0.094	0.072	T500P	0.032	0.069	0.049
R346S	0.078	0.076	0.069	T500Q	0.035	0.048	0.045
R346V	0.070	0.084	0.067	T500R	0.060	0.037	0.036
S494D	0.108	0.091	0.077	T500V	0.034	0.053	0.045
S494G	0.008	0.087	0.061	T500W	0.046	0.034	0.024
S494H	0.107	0.091	0.086	T500Y	0.052	0.032	0.016
S494I	0.081	0.090	0.090	V445A	0.081	0.091	0.094
S494K	0.171	0.105	0.158	V445E	0.094	0.083	0.069
S494N	0.051	0.088	0.058	V445F	0.050	0.074	0.070
S494P	0.088	0.085	0.062	V445I	0.008	0.081	0.065
S494T	0.034	0.087	0.080	V445Q	0.080	0.077	0.076
S494Y	0.123	0.093	0.119	V445R	0.095	0.072	0.060
T500A	0.035	0.061	0.040	V445T	0.067	0.089	0.092
T500C	0.037	0.040	0.026	V445W	0.094	0.068	0.072
T500D	0.078	0.051	0.034	Y449E	0.097	0.079	0.056
T500F	0.055	0.033	0.020	Y449G	0.029	0.074	0.074
T500H	0.046	0.037	0.029	Y449L	0.111	0.091	0.083
T500I	0.034	0.040	0.027	Y449M	0.064	0.098	0.089
T500K	0.062	0.033	0.028	Y449N	0.074	0.076	0.071
T500N	0.043	0.033	0.032	Y449P	0.121	0.079	0.074
T500S	0.007	0.049	0.025	Y449R	0.135	0.106	0.091
V445C	0.057	0.087	0.086	Y449S	0.064	0.084	0.085
V445D	0.115	0.097	0.089	Y449W	0.035	0.091	0.073
V445G	0.139	0.095	0.101	Y473F	0.008	0.097	0.086
V445H	0.058	0.066	0.063	Y473Q	0.134	0.141	0.175
V445K	0.112	0.065	0.072	Y473T	0.152	0.145	0.165
V445L	0.043	0.074	0.057	Y473V	0.128	0.141	0.141
V445M	0.023	0.069	0.057	Y473W	0.010	0.093	0.081
V445N	0.093	0.090	0.079	Y505E	0.000	0.011	0.019
V445P	0.124	0.093	0.102	Y505G	0.000	-0.044	0.011
V445S	0.093	0.089	0.083	Y505L	0.000	0.013	0.029
V445Y	0.096	0.070	0.069	Y505P	0.000	-0.022	0.015
Y449A	0.067	0.073	0.075	Y505R	0.000	0.052	0.025
Y449C	0.048	0.078	0.069	Y505S	0.000	-0.044	0.015
Y449D	0.111	0.082	0.079	Y505T	0.000	-0.044	0.014

Y449F	0.028	0.090	0.071	Y505V	0.000	-0.024	0.021
Y449H	0.044	0.083	0.062	Y505W	0.033	0.065	0.039
Y449I	0.097	0.091	0.090				
Y449K	0.184	0.142	0.147				
Y449Q	0.091	0.088	0.090				
Y449T	0.095	0.081	0.079				
Y449V	0.068	0.084	0.083				
Y473A	0.163	0.147	0.153				
Y473E	0.150	0.153	0.147				
Y473I	0.139	0.141	0.129				
Y473M	0.057	0.097	0.112				
Y473N	0.174	0.143	0.158				
Y473S	0.159	0.148	0.165				
Y489F	0.052	0.118	0.097				
Y489H	0.173	0.149	0.182				
Y489I	0.266	0.241	0.297				
Y489M	0.208	0.186	0.224				
Y489Q	0.239	0.212	0.274				
Y489V	0.256	0.232	0.269				
Y505A	0.000	-0.039	0.012				
Y505D	0.000	-0.026	0.009				
Y505F	0.007	0.075	0.047				
Y505H	0.009	0.029	0.029				
Y505I	0.000	-0.023	0.012				
Y505K	0.000	-0.002	0.007				
Y505M	0.015	0.011	0.026				
Y505N	0.011	-0.028	0.020				
Y505Q	0.018	-0.024	0.023				

Table S4. The experimental and predicted pK_D values of RBD variants with multiple mutations by MB-QSAR models.

Clone	Mutations	K_D (pM)	Relative pK_D[a] (Exp)	Relative pK_D (CoMFA)	Relative pK_D (CoSIA)
RBD-Wt	Residues 336–528	1,700	0.000	-0.204	-0.282
RBD-31	I358F, N448S, N501Y	907	0.273	-0.002	0.087
RBD-310	I358F, N448S, E484K, N501Y	607	0.447	1.057	0.761
RBD-312	I358F, V483E, N501Y	442	0.585	-0.129	0.182
RBD-316	I358F, N448S, E484K, F490S, N501Y	722	0.372	1.012	0.776
RBD-32	I358F, S477N, N501Y	348	0.689	0.881	0.909
RBD-33	I358F, E484K, N501Y	204	0.921	1.01	0.688
RBD-34	I358F, I468T, E484K, N501Y	644	0.422	1.012	0.763
RBD-36	I358F, I468T, N481Y, N501Y	268	0.802	-0.026	0.087
RBD-41	I358F, S477N, E484K, N501Y	104	1.213	1.859	1.547
RBD-47	I358F, N460K, E484K, N501Y	96	1.248	1.168	0.755
RBD-477KRY	I358F, S477R, E484K, Q498R, N501Y	30	1.753	1.588	2.132
RBD-477RY	I358F, S477N, Q498R, N501Y	46	1.568	0.919	1.577
RBD-478RY	I358F, T478I, Q498R, N501Y	93	1.262	-0.103	0.86
RBD-48	I358F, E484K, Q498R, N501Y	55	1.490	1.033	1.372
RBD-51	I358F, N460K, S477N, E484K, Q498R, N501Y	11	2.189	2.045	2.180
RBD-52	I358F, N460K, E484K, S494P, Q498R, N501Y, A520G	40	1.628	1.185	1.162
RBD-520	I358F, I468V, E484K, Q498R, N501Y	31	1.739	1.083	1.387
RBD-521	I358F, N460K, E484K, Q498R, N501Y	14	2.084	1.145	1.377
RBD-53	I358F, N460K, T478S, E484K, Q498R, N501Y	22	1.888	1.084	1.416
RBD-57	I358F, G446R, E484K, F490Y, Q498R, N501Y	35	1.686	1.761	2.099
RBD-610	I358F, N460K, S477N, T478S, E484K, S494P, Q498R, N501Y,	10	2.230	1.975	1.951

	S514T				
RBD-611	I358F, N460K, S477N, E484K, S494P, Q498R, N501Y, S514T	26	1.815	2.011	1.908
RBD-614	I358F, V445K, N460K, E484K, S494P, Q498R, N501Y	21	1.908	1.995	1.928
RBD-62	I358F, V445K, N460K, I468T, T470M, S477N, E484K, Q498R, N501Y	3.5	2.686	2.857	2.965
RBD-71	I358F, V367W, R408D, K417V, V445K, N460K, I468T, T470M, S477N, E484K, Q498R, N501Y	8.5	2.301	3.095	3.575
RBD-BRY	I358F, K417T, E484K, Q498R, N501Y	84	1.306	1.356	1.929
RBD-MBRY	I358F, K417T, T470M, E484K, Q498R, N501Y	56	1.482	1.404	1.950
RBD-RY	I358F, Q498R, N501Y	65	1.418	0.045	0.752
RBD-RYM	I358F, T470M, E484K, Q498R, N501Y	39	1.639	1.087	1.362
RBD-SARY	I358F, K417N, E484K, Q498R, N501Y	68	1.398	1.025	1.733

[a] $\Delta \log_{10} K_D$

Table S5. The relative pK_D of SARS-CoV-2 Omicron variants bound to hACE2

WHA label	Mutations on RBD	Experimental relative pK_D	Predicted relative pK_D	Predicted average escape fractions
BA.2.86	G339H, K356T, S371F, S373P, S375F, T376A, R403K, D405N, R408S, K417N, N440K, V445H, G446S, N450D, L452W, N460K, S477N, T478K, N481K, E484K, F486P, Q498R, N501Y, Y505H	1.05	4.82	0.57
BA.4/5	G339D, S371F, S373P, S375F, T376A, D405N, R408S, K417N, N440K, L452R, S477N, T478K, E484A, F486V, Q493R, Q498R, N501Y, Y505H	0.63	4.19	0.51
BQ.1.1	G339D, R346T, S371F, S373P, S375F, T376A, D405N, R408S, K417N, N440K, K444T, L452R, N460K, S477N, T478K, E484A, F486V, Q498R, N501Y, Y505H	0.73	3.56	0.54
EG.5	G339H, R346T, L368I, S371F, S373P, S375F, T376A, D405N, R408S, K417N, N440K, V445P, G446S, F456L, N460K, S477N, T478K, E484A, F486P, F490S, Q498R, N501Y, Y505H	0.35	2.60	0.62
HK.3	G339H, R346T, L368I, S371F, S373P, S375F, T376A, D405N, R408S, K417N, N440K, V445P, G446S, L455F, F456L, N460K, S477N, T478K, E484A, F486P, F490S, Q498R, N501Y, Y505H	0.96	3.98	0.60
JN.1	G339H, K356T, S371F, S373P, S375F, T376A, R403K, D405N, R408S, K417N, N440K, V445H, G446S, N450D, L452W, L455S, N460K, S477N, T478K, N481K, E484K, F486P, Q498R, N501Y, Y505H	0.16	2.15	0.64
JN.1.23	G339H, R346T, K356T, S371F, S373P, S375F, T376A, R403K, D405N, R408S, K417N, N440K, V445H, G446S, N450D, L452W, L455S, N460K, S477N, T478K, N481K, E484K, F486P, Q498R, N501Y, Y505H	0.92	4.17	0.62
JN.1.23.A475V	G339H, R346T, K356T, S371F, S373P, S375F, T376A, R403K, D405N, R408S, K417N, N440K, V445H, G446S, N450D, L452W, L455S, N460K, A475V, S477N, T478K, N481K, E484K, F486P, Q498R, N501Y, Y505H	0.24	2.27	0.60
JN.1.F456L	G339H, K356T, S371F, S373P, S375F, T376A, R403K, D405N, R408S, K417N, N440K, V445H, G446S, N450D, L452W, L455S, F456L, N460K, S477N, T478K, N481K, E484K, F486P, Q498R, N501Y, Y505H	0.20	1.64	0.71

JN.4	G339H, K356T, S371F, S373P, S375F, T376A, R403K, D405N, R408S, K417N, N440K, V445H, G446S, N450D, L452W, N460K, A475S, S477N, T478K, N481K, E484K, F486P, Q498R, N501Y, Y505H	0.35	2.52	0.55
JN.4.2	G339H, K356T, S371F, S373P, S375F, T376A, R403K, D405N, R408S, K417N, N440K, V445H, G446S, N450D, L452W, N460K, A475V, S477N, T478K, N481K, E484K, F486P, Q498R, N501Y, Y505H	0.35	2.59	0.54
KP.2	G339H, R364T, K356T, S371F, S373P, S375F, T376A, R403K, D405N, R408S, K417N, N440K, V445H, G446S, N450D, L452W, L455S, F456L, N460K, S477N, T478K, N481K, E484K, F486P, Q498R, N501Y, Y505H	0.24	2.04	0.69
KP.3	G339H, K356T, S371F, S373P, S375F, T376A, R403K, D405N, R408S, K417N, N440K, V445H, G446S, N450D, L452W, L455S, F456L, N460K, S477N, T478K, N481K, E484K, Q493E, F486P, Q498R, N501Y, Y505H	0.44	2.34	0.65
XBB.1.5	G339H, R346T, L368I, S371F, S373P, S375F, T376A, D405N, R408S, K417N, N440K, V445P, G446S, N460K, S477N, T478K, E484A, F486P, F490S, Q498R, N501Y, Y505H	0.50	3.72	0.57
